# Low-voltage electrohydraulic actuators for untethered robotics

**DOI:** 10.1126/sciadv.adi9319

**Published:** 2024-01-05

**Authors:** Stephan-Daniel Gravert, Elia Varini, Amirhossein Kazemipour, Mike Y. Michelis, Thomas Buchner, Ronan Hinchet, Robert K. Katzschmann

**Affiliations:** ^1^ Soft Robotics Lab, D-MAVT, ETH, Zurich, Switzerland.; ^2^ Computational Robotics Lab, D-INFK, ETH, Zurich, Switzerland.

## Abstract

Rigid robots can be precise but struggle in environments where compliance, robustness to disturbances, or energy efficiency is crucial. This has led researchers to develop biomimetic robots incorporating soft artificial muscles. Electrohydraulic actuators are promising artificial muscles that perform comparably to mammalian muscles in speed and power density. However, their operation requires several thousand volts. The high voltage leads to bulky and inefficient driving electronics. Here, we present hydraulically amplified low-voltage electrostatic (HALVE) actuators that match mammalian skeletal muscles in average power density (50.5 watts per kilogram) and peak strain rate (971% per second) at a 4.9 times lower driving voltage (1100 volts) compared to the state of the art. HALVE actuators are safe to touch, are waterproof, and exhibit self-clearing properties. We characterize, model, and validate key performance metrics of our actuator. Last, we demonstrate the utility of HALVE actuators on a robotic gripper and a soft robotic swimmer.

## Teaser

Reducing voltages in electrohydraulic actuators using ferroelectrics enables implementation in untethered biomimetic robots.

## INTRODUCTION

### Problem addressed

Rigid robots excel at precision and repetitive tasks but typically fall short in environments where compliance, robustness to disturbances, or energy efficiency is crucial (*1*). Therefore, researchers aim to mimic natural organisms, which are well adapted to operate effectively in unstructured environments (*2*). The musculoskeletal architecture evident in nature has inspired roboticists to widen their design space and create artificial muscles that mimic their natural counterparts for easier integration into wearables and untethered biomimetic robots.

Electromagnetic motors have been used to mimic musculoskeletal designs by placing them with tendons outside joints—for example, the Kengoro humanoid (*3*) or robotic hands (*4*–*6*). Although electromagnetically actuated tendon-driven robots show many degrees of freedom, they are inherently rigid and lack the desired dexterity, adaptability, softness, and efficiency found in natural musculoskeletal organisms. In contrast, soft fluidic actuators are inherently more compliant and require less energy in static load scenarios than electromagnetic actuators (*7*). For instance, McKibben pneumatic artificial muscles have high force density and can achieve strain values between 40 and 300% (*8*), which is more than the 20 to 40% strain of skeletal muscles (*9*). However, implementing soft fluidic actuators into untethered robots is challenging because of the need for pneumatic supply lines, pressure valves, and compressors (*10*).

Electrostatic actuators, such as dielectric elastomer actuators (DEAs), directly convert electrical energy into mechanical energy through deformations caused by electrostatic forces within the actuator (*11*). They can be inherently compliant and offer self-sensing capabilities (*12*). Owing to these beneficial properties, many muscle-like electrostatic actuators have been proposed (*12*–*18*), of which hydraulically amplified self-healing electrostatic (HASEL) actuators have emerged as promising candidates. HASEL actuators are electrohydraulic actuators that combine the features of soft fluidic actuators with electrostatic principles (*13*). These actuators are pouches filled with liquid and partially covered with electrodes. A voltage causes the electrodes to zip together, squeezing away the encapsulated liquid, thereby deforming the pouch and leading to a contraction of the actuator. Several HASEL actuator designs demonstrate strains ranging from 15 to 24%, blocking forces between 18 and 45 N (*9*, *19*), and a peak specific power on par with mammalian muscle (*12*). In general, the fabrication process of HASEL actuators is more straightforward than for DEAs (*13*); HASEL actuators can be made from biodegradable materials (*20*) and do not require prestretching or rigid frames to achieve high-strain and high-power operation (*12*).

HASEL actuators show promising performance metrics but are currently difficult to implement into complex untethered soft machines. It is primarily their high driving voltages (>6 kV) that require bulky power supplies with limited use in untethered applications. In addition, exposed electrodes are a key safety concern and limit the use of HASEL actuators in proximity to humans. Recent HASEL actuator designs made from thermoplastics do not show self-healing properties (*12*), which lowers system reliability.

### Objective

Many independent, easy-to-implement actuation channels are required to develop autonomous robots that mimic musculoskeletal organisms. We aim to develop a suitable, compliant artificial muscle system, including the actuator and driving electronics. This system should be small, safe, fast, powerful, and efficient. Decreasing the operating voltage of HASEL actuators is a key step to enabling high-performance, untethered soft robots. An approach to reducing voltages of HASEL actuators also has the potential to be applied to other electrohydraulic actuators (*16*, *17*, *21*–*26*).

### Background and related work

The required actuation voltage of HASEL actuator designs can be decreased by reducing the thickness or by increasing the permittivity of the dielectric material (*19*, *27*–*29*). Flexible polymers with high permittivity like polyvinylidene fluoride (PVDF) terpolymers have been successfully used to achieve high Maxwell stresses [>70 mN cm^−2^ V^−1^ (*30*)] at low voltages (<300 V) in compact electrostatic devices for haptic applications (*30*–*33*). For a Peano-HASEL actuator, a PVDF copolymer, poly(vinylidene fluoride-co-hexafluoropropylene) (PVDF-HFP), can enhance load-strain capability at a voltage of 5 kV (*34*). To achieve this capability, the patent reports a multilayered structure where different layers can be independently selected for their electrical and physical properties (*34*). High-k dielectrics, i.e., PVDF-HFP, have also been used to reduce threshold voltages in stretchable electroluminescent devices (*35*, *36*).

Another way of creating untethered systems with many actuation channels was to maintain a high switching voltage while optimizing the driving circuits. Mitchell *et al.* (*37*) developed a smartphone-sized (223 cm^3^, 250 g) 10-channel 10-kV unipolar power supply. They used high-voltage optocouplers, which allowed them to reduce the power supply in terms of size, but at the cost of efficiency (estimated 7% at low load and 37% at maximum load) and with a high price (1200 USD per power supply unit). Moreover, driving circuits based on high-voltage optocouplers consume, while idling, high amounts of energy to ground the actuators (approximately 0.25 W per actuator channel); this power consumption makes them unpractical for untethered robots.

A strategy for reducing the size, complexity, and cost of the driving electronics (*37*) is to design electrostatic actuators that operate at approximately a few hundred volts. For instance, a subgram robotic insect was developed by reducing the required driving voltages of its miniature DEA to <500 V to allow for tiny power electronics (millimeter scale) (*38*). Many low-cost electronic components operating in hundreds of volts are available to serve the established field of piezoelectric devices (*37*).

Metal-oxide semiconductor field-effect transistors (MOSFETs), such as the IPN95R3K7P7m by Infineon, can operate at around 1 kV. Compared to optocouplers, this MOSFET provides a smaller volume (three times lower), at a lower price (two orders of magnitude cheaper), and with reduced power consumption (two orders of magnitude lower) (see table S1). The rapid switching speed of MOSFETs (on the order of nanoseconds) compared to optocouplers (on the order of microseconds) and their higher output current (2 A versus 0.5 mA) hold promise for advancing real-time control in HASEL actuators. Despite these advantages, MOSFETs are limited to switching voltages below 5 kV, while current HASEL actuators require above 5 kV for sufficient performance. In addition, the footprint and price of commercially available MOSFETs decrease by an order of magnitude when transitioning from devices operating in the thousands of volts to those operating in the hundreds of volts (see fig. S1 for an overview).

### Contributions

**Fig. 1.**
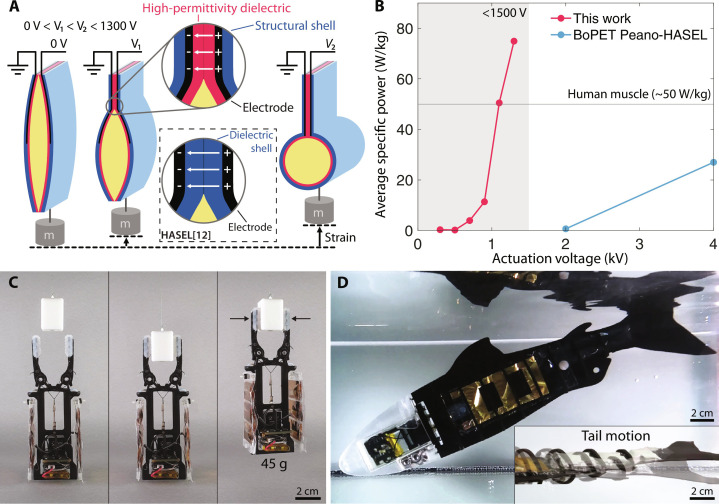
A low-voltage muscle system for untethered electrostatic robots. (**A**) General working mechanism of the actuator and detailed view of the composite film structure. Three stages show the actuator in a not-contracted, slightly zipped, and fully zipped state. The transition from relaxed to fully zipped can be seen with 0 < *V*_1_ < *V*_2_. The zoomed-in view shows the components of the composite film structure: structural load-carrying shell, electrode, and high permittivity dielectric. As a comparison, a second zoom-in is shown for the Peano-HASEL structure. (**B**) Average specific power of a HALVE actuator (5-μm PVDF-TrFE-CTFE) versus a Peano-HASEL actuator (15-μm BoPET as the dielectric shell) tested for a 300-g load. The horizontal line indicates the typical specific power of mammalian skeletal muscle at 50 W kg^−1^ (*39*). (**C**) Untethered gripper weighing 45 g can grasp a smooth plastic block with a pinch grasp firmly enough to be lifted into the air. (**D**) Fully integrated demonstrator artificial fish floating in the water. The artificial fish measures approximately 28 mm in length.

This paper contributes, to the field of robotics, a muscle-like, contracting actuator with a 4.9 to 6.6 times lower driving voltage at equal force-strain performance than other electrohydraulic actuator designs that use linear dielectrics. On the basis of the idea of a multilayer structure for a Peano-HASEL actuator (*34*), we chose as a force-bearing layer polyethylene terephthalate (PET) and as a force-producing layer the relaxor-ferroelectric PVDF terpolymer (see Fig. 1A). The thin layer of poly(vinylidene fluoride-trifluoroethylene-chlorotrifluoroethylene) or short P(VDF-TrFE-CTFE) increases the permittivity and, therefore, reduces the actuator’s driving voltages. The surrounding force-bearing layer ensures the strength and encapsulation of the electrode.

Our hydraulically amplified low-voltage electrostatic (HALVE) actuator achieves an average power density of 50.5 W kg^−1^ and peak strain rate of 971% s^−1^ comparable to mammalian skeletal muscle (typical 50 W kg^−1^, peak 500% s^−1^) (*39*–*42*) at an actuation voltage of 1100 V (see Fig. 1B). In addition, HALVE actuators are safe to touch, waterproof, and exhibit self-clearing properties, making them easy to implement in robotics or wearables. The self-clearing property enables the actuator to endure multiple dielectric breakdowns while remaining operational.

We provide extensive experimental characterizations of the HALVE actuators and comparisons with state-of-the-art Peano-HASEL actuator designs for key performance metrics. To accurately predict our actuators’ force output, we modeled and physically validated the electrostatic force with the energy density of the relaxor-ferroelectric material. Last, we integrate HALVE actuators into a functional muscle system that includes a small, custom-made, and efficient power supply, creating a gripper (see Fig. 1C) and a robotic fish (see Fig. 1D) to show the potential of this technology for untethered robotic applications.

## RESULTS

### Actuator overview

In HASEL actuators (*13*), a thin thermoplastic film, typically either biaxially oriented polypropylene (BOPP) or biaxially oriented PET (BoPET), serves two functions: (i) as the actuator’s structural element and (ii) as the dielectric layer for the electrostatic actuation. This functional duality constrains the choice of material because the material cannot be optimized for high tensile strength and dielectric properties independently. Electrohydraulic actuators could be further optimized by decoupling these two functions into separate elements of the actuator structure (*34*), allowing for more specific material selection.

We use a different device structure (see Fig. 1A) composed of three elements. The first element is an outer shell made of a mechanically strong polymer that serves as the structural element and as an outer electric insulation layer that covers the entire device to avoid electrical discharge to the environment; the second element is an electrode that covers a portion of the pouch; and the third one is a thin layer of P(VDF-TrFE-CTFE) that serves as a high-energy density dielectric layer to boost electrostatic actuation performance and lower the actuation voltage. Last, the central cavity is filled with dielectric oil, which functions as the actuator’s hydraulic amplification medium.

We use the Peano-HASEL actuator geometry (*12*) to study the capabilities of the three-layer material composite structure at voltages below 1300 V. We call the resulting actuator a HALVE actuator. The principle of operation is the same as for Peano-HASELs (*12*): When a voltage difference is applied between the electrodes, opposite electric charges build up in each electrode. The resulting Coulomb force attracts the electrodes that squeeze away the dielectric oil to the bottom part of the pouch, progressively deforming into a cylindrical shape. This change in geometry shortens the length of the actuator, thus generating mechanical work (see Fig. 1A).

### Model

**Fig. 2.**
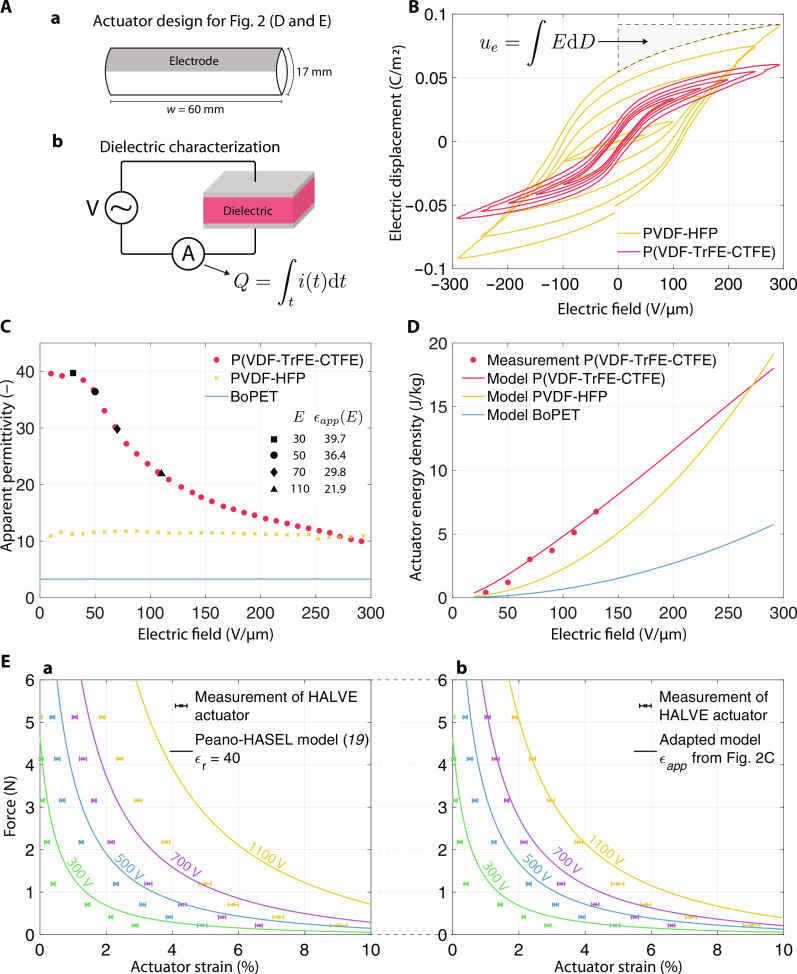
Apparent dielectric constant for model predictions of HALVE actuators force/strain behavior and model validation. (**A**) (a) Actuator design specifications for which the actuator energy densities and force/strain curves are calculated and measured in (D) and (E). (b) Depiction of the dielectric measurement setup used to measure the data points for (B). (**B**) Discharge *D*-*E* curves for PVDF-HFP and P(VDF-TrFE-CTFE), the highlighted gray area corresponds to the energy density integral of PVDF-HFP at 300 V μm^−1^. (**C**) Apparent permittivities calculated using Eq. 5 with the experimental data shown in (B). (**D**) Prediction and measurement for the resulting actuator energy densities for different dielectrics. (**E**) Force/strain measurements and model prediction. (a) Model is plotted with a constant permittivity of 40, as suggested by Kellaris *et al.* (*19*). (b) Model is plotted with apparent dielectric constant values from (C).

Kellaris *et al.* (*19*) have proposed a quasi-static model of Peano-HASEL actuators. Assuming that the mechanical energy stored in the actuator is negligible, the total free energy *U_t_* of the system is

$$U_{t}=\mathrm{Fx}-U_{e}$$ (1)

where *F* is the mechanical force generated by the actuator over the distance *x*. The product of *F* and *x* represents the mechanical work produced by the actuator. For a given design, the free energy is constant once it is at equilibrium. To increase the mechanical work, the electrical energy *U_e_* must be increased, which is given by

$$U_{e}=\frac{1}{2}CV^{2}$$ (2)

where *C* is the capacitance between the actuator’s electrodes, and *V* is the voltage applied between the electrodes. The voltage *V* can be raised to increase the electrical energy, but keeping it as low as possible for wearable and autonomous applications is advantageous. Instead, we want to increase the capacitance *C*. Considering the zipped region of a Peano-HASEL actuator as an ideal parallel plate capacitor with a homogeneous polymer insulating layer and neglecting the oil thickness remaining at the interface, the capacitance can be modeled as

$$C=\frac{A\epsilon_{0}\epsilon_{r}}{t}=\frac{\mathrm{wl}\epsilon_{0}\epsilon_{r}}{t}$$ (3)

where *A* is the zipped area of the electrodes with length *l* and width *w*, *t* is the thickness of the dielectric, ϵ_0_ the vacuum permittivity, and ϵ_r_ is the relative permittivity of the insulator. The dimensions of the actuators used in all subsequent characterizations are illustrated in Fig. 2A (a).

We select a flexible insulator with higher permittivity, which can be a PVDF copolymer such as PVDF-HFP or terpolymer (PVDFter) such as P(VDF-TrFE-CTFE) (*30*), to increase the capacitive electrical energy for a given voltage and design. According to Eqs. 2 and 3, replacing the commonly used 15-μm-thick BoPET linear dielectric film (*14*) (ϵ_r_ = 3.3) with a 5-μm-thick P(VDF-TrFE-CTFE) nonlinear dielectric layer (ϵ_r_ = 40) could theoretically reduce the required operating voltage by approximately six times. However, it is more complex in practice because the permittivity of such high-permittivity insulators is not constant but depends on many factors, such as the amplitude and frequency of the applied electric field and the temperature (*43*). In addition, the fabrication process and material quality affect the insulator’s permittivity.

We model nonlinear dielectric materials, such as PVDF-HFP and P(VDF-TrFE-CTFE), using the system’s electrical energy. The energy density of a dielectric (*44*) is given by

$$u_{e}=\int ‍EdD$$ (4)

where *D* is the electric displacement and *E* is the amplitude of the electric field. Multiple *D*-*E* bipolar hysteresis curves of PVDF-HFP and P(VDF-TrFE-CTFE) were recorded using the measurement setup shown in Fig. 2A (b). The resulting hysteresis curves are displayed in Fig. 2B for different field strength amplitudes. The integral of the *D*-*E* bipolar discharge curve for one measurement is highlighted in Fig. 2B. As a quantitative estimation of the nonlinearity of ferroelectrics, Chu *et al.* (*44*) propose to model the energy density as a function of the applied electric field amplitude. We call the parameter for the quantitative estimation the apparent permittivity ϵ*_app_*(*E*), which is derived from Eq. 4 according to the generalized mean value theorem for integrals

$$u_{e}=\int ‍EdD=\frac{1}{2}\epsilon_{0}\epsilon_{\mathrm{app}}\left(E\right)E^{2}$$ (5)

The apparent permittivity, therefore, represents the mean slope of the discharge *D*-*E* hysteresis curve (see fig. S2). Figure 2C displays the apparent permittivities calculated using Eq. 5. Measurements showed that at low frequencies (2 Hz), the apparent permittivity of PVDF-HFP is almost constant for electric fields up to 300 V μm^−1^. However, the measured apparent permittivity of the relaxor-ferroelectric P(VDF-TrFE-CTFE) peaked at 39.5 at 30 V μm^−1^ and then decreased to 10 at 300 V μm^−1^ (see Fig. 2C). This behavior is similar to the one reported by Chu *et al.* (*44*). These measurements suggested that above 300 V μm^−1^, PVDF-HFP has a higher apparent permittivity than P(VDF-TrFE-CTFE).

Kellaris *et al.* (*19*) derived the parametrized force-strain relationship of the Peano-HASEL actuator from its total free energy (see Eq. 1). To approximate a model for nonlinear dielectrics, we swap the relative dielectric constant ϵ_r_ with the apparent dielectric constant ϵ*_app_*(*E*)

$$F=\mathrm{wt}\frac{\text{cos}\left(\alpha \right)}{1-\text{cos}\left(\alpha \right)}\epsilon_{0}\epsilon_{\mathrm{app}}\left(E\right)E^{2}$$ (6)

where α is the opening angle of the pouch, *w* is the width of the Peano-HASEL, and *t* is the thickness of the dielectric as defined in (*19*). The strain of the Peano-HASEL actuator can be calculated using the opening angle of the pouch α as shown in (*19*). For a HALVE actuator, *t* corresponds to the thickness of the high–energy density dielectric layer between the electrode and the oil. The adapted force-strain equation allowed us to predict actuator energy densities by integrating the area under the modeled force-strain curve divided by the weight of the actuator (*18*). We used Eq. 6 and the values from Fig. 2C to calculate the theoretical actuator energy densities of HALVE devices. Figure 2D shows the predicted actuator energy densities of an actuator made of P(VDF-TrFE-CTFE), PVDF-HFP, or BoPET as dielectric using Eq. 6 and the values from Fig. 2C. We can see that for P(VDF-TrFE-CTFE), the relationship between actuator energy density (force of the actuator) and the applied electric field is nearly linear, whereas for PVDF-HFP and BoPET, it is quadratic. This almost linear force response of PVDF terpolymers to applied voltages has been previously noted in the literature (*44*–*46*). We observed that an actuator made from PVDF-HFP achieves a higher actuator energy density above 280 V μm^−1^, owing to a drop of the dielectric energy density of P(VDF-TrFE-CTFE) below the value of PVDF-HFP under high electric fields. Figure 2E shows an increasing discrepancy with higher voltages between the theoretical force-strain curves of HALVE devices calculated considering a constant permittivity of 40 on the left (a) and using the apparent permittivity ϵ*_app_* of P(VDF-TrFE-CTFE) on the right (b).

### Performance characterization

**Fig. 3.**
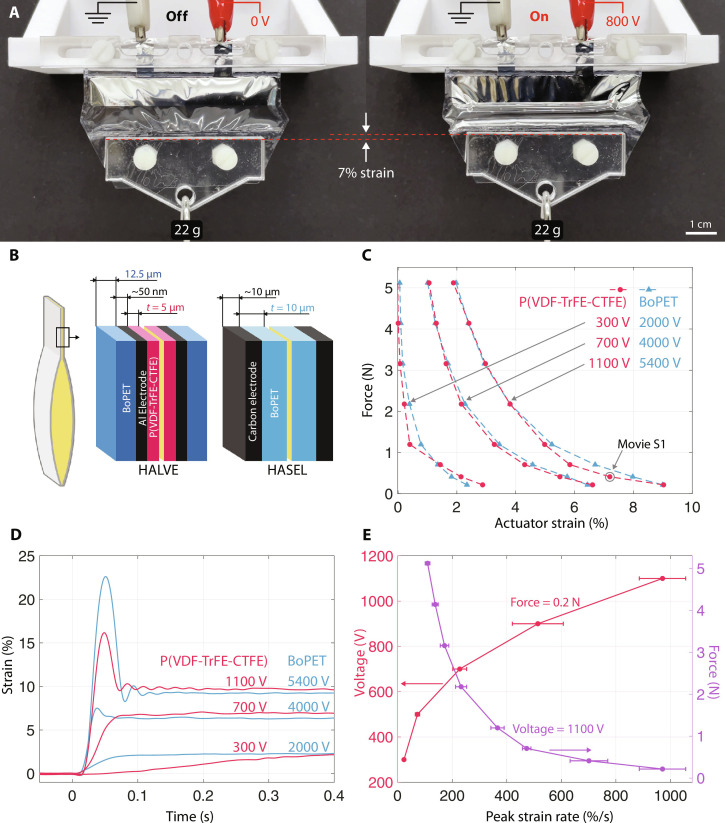
Performance characterization of the HALVE actuator. (**A**) HALVE actuator at rest and contracted during actuation. (**B**) Composite structures of the HALVE and Peano-HASEL actuators used for the experiments in (C) to (E). *t* indicates the dielectric layer thickness. (**C**) Strain-force curves of a *t* = 5-μm P(VDF-TrFE-CTFE) HALVE actuator and a *t* = 15-μm BoPET Peano-HASEL actuator, in pairs of actuation voltages that result in similar performance. (**D**) Strain response to step inputs of different voltage amplitudes for a *t* = 5-μm P(VDF-TrFE-CTFE) HALVE actuator and a *t* = 15-μm BoPET Peano-HASEL actuator lifting 22-g weights. (**E**) Left axis: the relationship between peak actuation strain rate and actuation voltage of a P(VDF-TrFE-CTFE) HALVE actuator at a constant force of 0.22 N. Right axis: the relationship between peak actuation strain rate and force at a constant voltage of 1100 V.

To validate the model, the quasi-static relationship between the actuation force and strain of HALVE actuators (see Fig. 2E) was experimentally characterized by measuring the displacement produced by the actuators at a given actuation voltage and loaded with a known weight attached to them (see fig. S3A). Figure 3A shows one of these strain measurements with a HALVE actuator lifting 22 g at 800 V by 7% strain. HALVE actuators were made from BoPET film as the structural shell, with a 5-μm-thick P(VDF-TrFE-CTFE) layer as the solid dielectric and thin aluminum electrodes (see Fig. 3B). The same electrode and pouch geometry (see Fig. 2A, a) was used in all characterizations. HALVE actuator devices were manufactured and measured following the process described in Materials and Methods.

The results of the characterization measurements are shown in Fig. 2E and are compared to the model prediction. Owing to its lower energy density at high electric fields, the model predicted that the force of a HALVE device actuated at 1100 V reaches approximately 5 N at only 2% strain instead of 4%. This prediction agrees with the measured values (see Fig. 2E). Still, both models overpredicted the performance at high strain, which could result from imprecise filling amounts and edge constraints. As expected, both models predicted similar performances at low electric fields because the relative and apparent dielectric constants have similar values.

To gain a more comprehensive understanding of the strengths and limitations of our approach, we compared HALVE actuators with Peano-HASEL actuators. Peano-HASEL actuators were manufactured from thermoplastic polymers, as suggested in the literature (*12*, *14*, *15*), with 15-μm-thick BoPET film as the solid dielectric layer with carbon ink electrodes (see Fig. 3B). HASEL and HALVE actuators had the same size and geometry (see Fig. 2A, a). The weights of both actuators were similar because they contained the same volume of oil and had nearly identical total film thicknesses.

To mitigate any charge retention in the dielectrics, we applied a voltage signal with an alternating polarity (bipolar) to both actuator types (*30*, *47*). We recorded the strain curves at various voltage amplitudes and weights for both HASEL and HALVE actuator devices. As expected, the force decreased with increasing strain (see Fig. 3C), following a profile in agreement with the literature (*19*) and with the datasheet of commercially available Peano-HASEL actuators (*48*). As shown in Fig. 3C, the quasi-static performance of HALVE actuators was comparable to that of HASEL actuators at a lower voltage between 4.9 and 6.6 times.

Movie S1 shows a HALVE device that was actuated at 1100 V and produced approximately 7% strain when lifting a 42-g weight. The P(VDF-TrFE-CTFE) layer of HALVE devices should be able to withstand field strengths of up to 350 V μm^−1^ (*30*). In practice, however, breakdowns occurred regularly above 120 V μm^−1^, potentially as a result of defects and impurities introduced during manufacturing outside a clean room. Reliability testing was performed at 800 V with a 200-g load over 2500 cycles (see fig. S3B.) We also tested charge retention (*49*) and charge accumulation performance by contracting an actuator for 60 s at 1100 V (see fig. S3C). Peano-HASEL actuators made from industrial-grade BoPET films were able to tolerate higher field strengths (>300 V μm^−1^) and, therefore, were able to outperform HALVE actuators’ force-strain characteristics when driven at voltages above 6 kV (see fig. S4).

To further validate the adapted model, we compared the model prediction for the actuator energy density with values derived from measured force-strain curves (see Fig. 2D). To determine the actuators’ energy density across the actuation range, we calculated the surface below the measured force-strain curve (*18*). For the integration of measured force-strain curves, we performed an optimized curve-fit onto the force-strain measurement data with a box-constrained optimization and system identification on eight relevant parameters using the analytical formula derived by Kellaris *et al.* (*19*). The optimization is explained in more detail in the Supplementary Materials. We observed good matching between the model prediction and the measured values for the actuator energy density (see Fig. 2D).

Actuation voltages also affect the dynamic response of HASEL actuators as reported in the literature (*12*, *47*). As shown in Fig. 3D, the higher the voltage applied to the actuators, the faster the actuation for the same force. For BoPET Peano-HASEL actuators, we observed that the strain slowly increased at 2000 V until reaching a stationary regime. At higher voltages, Peano-HASEL actuators show under-damped oscillations and strain overshoot, as reported in (*12*). We observed the same phenomena with HALVE devices but with more damping because the voltages involved were lower. The strain rate is proportional to the square of the voltage and inversely proportional to the actuation force (see Fig. 3E). The strain rate for HALVE actuators was 971% s^−1^ at 1100 V, lifting a 22-g weight, which is comparable to the maximum strain rate achieved for BOPP Peano-HASEL actuators (*12*) and about twice the maximum strain rate in mammalian skeletal muscle (500% s^−1^) (*12*, *40*). Other parameters can influence the strain rate of HASEL actuators; analytical models (*47*) predict that the strain rate depends on the geometry of the actuator and viscosity of the dielectric liquid. Those parameters could be optimized to improve the strain rate of HALVE actuators.

Average and peak specific powers were calculated during contraction cycles following the same steps as outlined by Kellaris *et al.* (see fig. S5) (*12*). The comparison of the average specific power of a HALVE and Peano-HASEL actuator is shown in Fig. 1B for a 300-g load. The HALVE actuator reached an average specific power of 50.5 W kg^−1^ at an actuation voltage of 1100 V and 75 W kg^−1^ at an actuation voltage of 1300 V. Peak and average specific power values for various loads and voltages are shown in fig. S6. The peak specific power in HALVE devices reached 132.7 W kg^−1^ at 1300 V for a load of 300 g, which is comparable to mammalian skeletal muscle ranging between 50 W kg^−1^ (typical) and 284 W kg^−1^ (maximum) (*39*). While our load versus specific power measurements (see fig. S6) follow the same trajectory reported by Kellaris *et al.* (*12*), we observed that our actuators achieve the highest specific power at a load of 300 g, which is higher than the corresponding result for BOPP Peano-HASEL actuators at a load of 100 g (*12*).

### System integration properties

#### Interaction with the environment

**Fig. 4.**
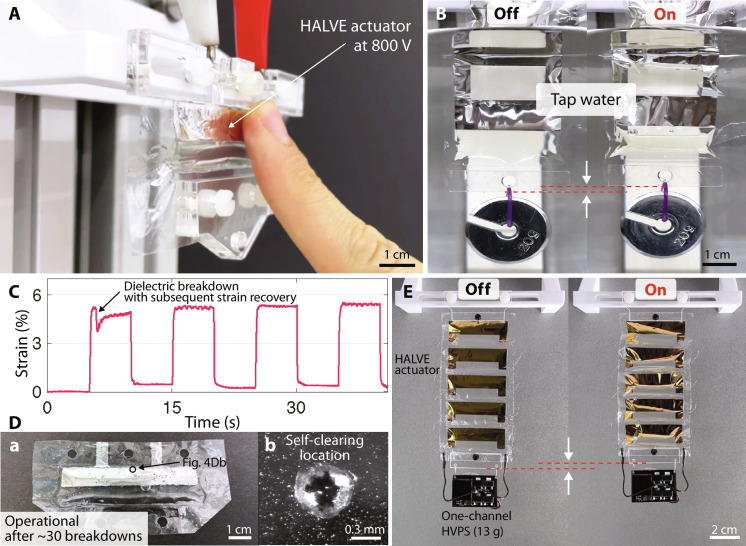
Implementation features of the HALVE actuator system. (**A**) A HALVE actuator is touched on the high-voltage side in the zipped state at 800 V. (**B**) A three-pouch HALVE actuator partially submerged in a laboratory beaker filled with tap water lifting a 20-g weight. (**C**) Strain curve of a single-pouch HALVE actuator lifting a 22-g weight at a low frequency of 0.1 Hz at 500 V. The actuator drops slightly in strain after the first dielectric breakdown event and recovers its full strain over the next few seconds. (**D**) Pictures of an actuator that experienced multiple dielectric breakdowns. (a) Picture after approximately 30 dielectric breakdown events, after which the actuator is still operational. (b) Zoomed picture of a self-clearing location. (**E**) Fully untethered actuator system, showing the compactness of the overall system. A five-pouch HALVE actuator with chrome/gold electrodes is lifting its power supply (13 g).

The outer structural shell of the HALVE actuator (see Fig. 1A) insulates the electrodes and allows the actuator to come into contact with the environment; the shell also protects the solid dielectric from humidity. Thus, the actuator can be touched during actuation (see Fig. 4A), and it works underwater (see Fig. 4B). Movie S2 shows a one-pouch HALVE actuator touched on the high-voltage electrode side while actuated at 800 V. Movie S3 shows a three-pouch HALVE actuator being actuated at 900 V while partially submerged in tap water. When a HALVE actuator is touched or operated underwater, the structural shell can act as a dielectric layer, which creates a second capacitance toward the outside of the actuator. This capacitor can interfere with the main capacitance between the actuator’s electrodes. However, this effect is typically negligible because the structural shell has a smaller permittivity and is thicker than the internal dielectric. Discharge is unlikely to happen toward the outside of the actuator because the BoPET structural shell has a very high breakdown voltage of 7500 V, much higher than the actuation voltages. Other methods have been proposed to use HASELs underwater using water as the ground electrode (*50*). However, the resulting actuator (*50*) only functions if the electronics and actuator are fully submerged, and it only allows for unipolar actuation, which increases charge retention.

#### Self-clearing

The self-clearing property can make HALVE actuators more resilient to manufacturing defects that can cause dielectric breakdowns. During a breakdown event, a HALVE actuator’s electrode is immediately destroyed locally, effectively self-clearing the short circuit. We observed that the outer shell remains largely undamaged because the shell is more robust than the internal dielectric, and the breakdown occurs internally between the two electrodes. An intact outer shell minimizes the risk of oil leaks from the inner pouch. This self-clearing feature allowed our HALVE actuators to withstand multiple breakdowns while remaining functional.

The impact of a dielectric breakdown at 500 V on the strain performance of a HALVE actuator is shown in Fig. 4C. Subsequent force-strain measurements of the same actuator are presented in fig. S7A, while fig. S7B depicts strain-time curves of subsequent dielectric breakdowns of this actuator. Self-clearing events can generate burned particles that can contaminate the oil and degrade the actuator’s performance. The effects of charge retention, resulting from oil impurities, are observable in fig. S7B. Figure 4D (a) shows this actuator after approximately 30 breakdowns while still functional. Figure 4D (b) shows a close-up image of one of the self-clearing areas. The main advantage of the self-clearing property is that a single breakdown event in one pouch typically does not short an entire multi-pouch actuator, which makes implementation, servicing, and prolonged usage of this actuator in untethered systems easier. For BoPET HASEL actuators, we observed that a single dielectric breakdown punches a hole into the oil-filled pouch, leaking the oil and typically shorting the electrodes by melting both zipped sides together.

#### Electronics and system size

We developed an autonomous high-voltage power supply based on the Peta-pico-Voltron design (*51*) to power the HALVE devices (see fig. S8). Figure 4E and movie S4 show a five-pouch HALVE actuator lifting one of these custom-developed power supplies, which provides 900 V to the actuator.

As core components, we used a miniature 1-kV DC/DC converter from HVM Technology and commercially available high-voltage (HV) MOSFETs (IRF7509TRPBF) (*V_DS_* ≤ 950 V). The module also includes a microcontroller, radio, or Bluetooth antenna, and a 150-mAh lithium polymer (LiPo) battery; the module can be extended with other components from the TinyCircuits ecosystem. The entire unit, excluding the DC/DC HV converter, requires 0.35 W of power. The DC/DC HV converter requires between 0.05 W at no load and 0.875 W at maximum load. The two-channel electronic assembly is 42 mm by 19 mm by 22 mm in size or 11 cm^3^ in volume and weighs 15.5 g. The one-channel variant used for the gripper weighs slightly less at 13 g. The assembly can easily be extended to more channels by repeating the H-bridge circuit (see fig. S9). Each additional channel adds approximately 1.4 cm in length or 1.5 cm^3^ in volume to the power supply.

In fig. S8C, we compare the frequency response of a 0.5-W version with that of a 0.1-W version and observe that a more powerful DC/DC converter can improve the actuators’ frequency response. A DC/DC converter with a higher power output can be used to maintain actuation speeds at similar levels when using a greater number of actuators or actuators with larger electrode areas (i.e., higher capacitance).

The price for the two-channel high-voltage power unit is approximately 150 USD for the DC/DC converter plus approximately 35 USD for all other components (excluding the microcontroller, communication module, and battery). As a comparison, a larger full-bridge 10-kV two-channel power supply with high-voltage optocouplers (*15*) is approximately 440 USD for the DC/DC converter and 760 USD for the eight required optocouplers at current market prices (August 2023).

### Untethered robotic demonstrators

#### Gripper

**Fig. 5.**
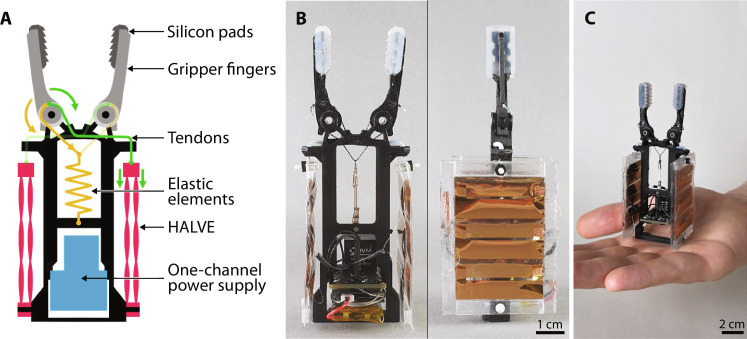
Untethered gripper demonstrator powered by the HALVE actuator. (**A**) Schematic view of the untethered gripper. The figure shows the tendon system that connects a HALVE actuator muscle pack to each gripper finger, as well as the return spring visible in green and yellow, respectively. Arrows of the same color mark the direction in which the tendon is pulled and the direction of the resulting torque on the finger’s joint. (**B**) Front and side views of the untethered gripper. (**C**) Size comparison of the HALVE actuator gripper to a human hand.

We demonstrate the potential of HALVE actuators with their small form factor control electronics with an untethered gripper powered by HALVE actuators (see Fig. 5A). Movie S5 shows the untethered gripper grasping a block printed from polylactic acid (PLA). The custom gripper comprises a main body to which two fingers are attached via revolute joints. The fingertips are equipped with cast silicone pads to improve the grip (see Fig. 5A). Two packs of HALVE muscles are used to generate gripping force. Each pack comprises two individual actuators connected in parallel and composed of three pouches in series. The actuator packs are fixed to the main body at one extremity, while the other extremity is linked to one of the gripping fingers via a tendon. An elastic element, connected to the fingers via another set of tendons, serves as an antagonistic element to the actuators, restoring the gripper to an open state after gripping. The entire gripper is powered by a single-channel power supply that delivers 900 V to the gripper’s actuators (see Fig. 5B). The power supply is driven by a 150-mAh LiPo battery (see Fig. 5B). The combined weight of the battery and power supply is 15 g. The entire gripper assembly weighs 45 g, including electronics, and has a volume of 112 mm (height) by 40 mm (width) by 45 mm (depth) (see Fig. 5C for a size comparison to a human hand). Figure 1C and movie S5 show how this untethered gripper can pinch grasp a smooth plastic (PLA) object firmly enough to carry its weight when the object is lifted with a string into the air.

#### Bioinspired swimmer

**Fig. 6.**
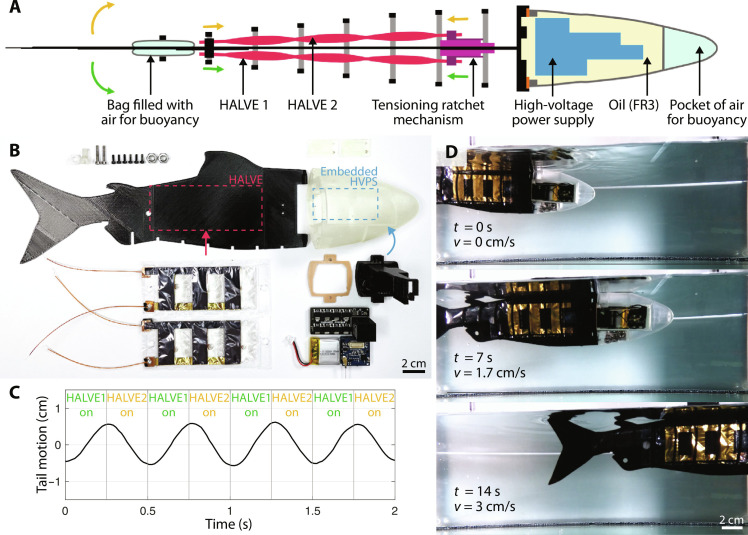
Untethered artificial fish for underwater locomotion. (**A**) The soft robotic fish assembly structure. The green and yellow arrows indicate the forces and resulting deformation generated by the HALVE actuators. (**B**) Components of the fish before assembly. Airbags for buoyancy and the ribs are not shown. (**C**) Left and right oscillating motion of the robotic fish tail measured at the tip. Also shown is the antagonistic activation of the HALVE actuators. (**D**) Swimming robotic fish accelerating from a stationary position to reach a speed of 3 cm s^−1^ after 14 s.

We validated and tested the integrated actuator system in a fully untethered, self-contained system, namely, the bioinspired swimmer in Fig. 6 (further component details shown in fig. S10). The robotic fish (28 cm in length) was composed of a head containing the electronics and a flexible body onto which the HALVE actuators were attached and tensioned (see Fig. 6A). The outer shell of the HALVE actuators for the fish was made from BoPVDF for improved seal performance. The head was made of a rigid, semitransparent watertight shell (see Fig. 6B) and was filled with dielectric oil (Envirotemp FR3) to protect the electronics from any water that might enter the cavity. The body was made of a flexible three-dimensionally (3D)–printed skeleton of PLA onto which the HALVE actuators were attached. A tensioning ratchet system was used to tighten the actuators individually to conform to any deviations in manufacturing. Air pouches balanced the fin and head, allowing the system to be a surface swimmer.

The shape and materials were modeled and simulated in COMSOL to predict the tail bending for the attachment points of the HALVE actuators. The actuators were simulated using a force lookup from the corresponding force-strain measurement curves. Those forces were applied on the attachment points of the HALVE actuators to simulate the system’s tail deformation. We found through our modeling that under no external loading, a tip deformation of up to 61.4 mm can be expected, which was compared to the experimental data (see fig. S11).

We prepared a 30 cm–by–35 cm–by–60 cm tank filled up to a height of 30 with room-temperature tap water. We placed the swimmer in the aquarium and actuated the HALVE actuators antagonistically at 800 V to observe forward thrust. Movie S6 shows the untethered artificial fish swimming in tap water with 2-Hz antagonistic actuation. We recorded the motion trajectory of the fishtail (see Fig. 6C) and measured the performance of the fish by its forward-swimming velocity. We observed a maximum swimming velocity of 3.8 cm s^−1^, equivalent to 0.14 body length/s at 2-Hz antagonistic actuation. Figure 6D shows three snapshots of the swimming fish after 0, 7, and 14 s with the corresponding speeds. The swimming velocity and acceleration for the first 10 s of a swim are shown in fig. S12. Aside from forward-swimming velocity, we also characterized the efficiency of our swimmer by measuring the power consumption of the high-voltage power supply during a 2-Hz antagonistic actuation, which was, on average, 0.6 W. On a full charge, the 0.54-W battery of the fish can last for approximately 54 min during continuous actuation.

## DISCUSSION

We have shown a promising actuator system—the HALVE actuator. Its key properties are its low-voltage characteristics, high power density, easy implementation, and high robustness (self-clearing). The three-layer design approach has enabled us to use high-energy density dielectrics, drastically reducing the voltage requirements of Peano-HASEL actuators to hundreds of volts. In some areas, such as peak strain rate and average specific power, HALVE actuators perform similarly to mammalian skeletal muscle. HALVE actuators have several desirable features for implementation, such as safe operation in proximity to humans (*52*) and the ability to operate in water. Owing to the small size and high efficiency of the required power supplies, these actuators can be easily scaled to multichannel systems. This compactness and efficiency stand in contrast to most electrostatic actuators, which are typically difficult to implement and scale in practice.

One challenge with the current design is the durability (see fig. S3B) of the heat seals, which typically fuse only the P(VDF-TrFE-CTFE) layers, not the outer structural shell. To address this problem, we developed a manufacturing method using BoPVDF as the outer shell for the robotic fish demonstrator. However, this method has drawbacks, including a substantial increase in material cost, required equipment, and manufacturing time and labor. Another challenge is the actuators’ relatively low force density, typical for electrohydraulic actuators. Although the average specific power of HALVE actuators is in the range of natural muscle (50 W kg^−1^), the maximum force density is currently lower. Force density could be improved by increasing the breakdown voltage of the thin P(VDF-TrFE-CTFE) films. The current manufacturing processes limited the quality of the thin dielectric films we fabricated. As a result, we experimentally observed that current HALVE actuators have a dielectric strength of around 120 V μm^−1^ only. The dielectric strength could be improved considering the P(VDF-TrFE-CTFE) literature and the manufacturer’s specifications. For example, moving manufacturing into clean rooms, filtering the casting solution, and reducing porosity and cracks in the dielectric with more precise temperature control during cross-linking could improve the quality of the dielectric films.

Moving forward, there are numerous opportunities for exploring the application of more sophisticated high-energy density dielectrics in HALVE actuators and electrohydraulic actuators more generally. Recently developed dielectrics such as [(Bi0.5Na0.5)TiO_3_–NaNbO_3_]/PVDF-HFP nanocomposites could further increase the power density, potentially outperforming mammalian skeletal muscle (*53*). Even smaller driving electronics, such as the ones shown by Ji *et al.* (*38*), would enable biologically inspired autonomous robots with many independent channels. Other manufacturing methods for depositing the dielectric to create higher-quality thin films with high breakdown strength should be investigated. One promising technology is electrospraying, which has been successfully used to create very thin high-quality dielectric films for DEAs (*54*). As it stands, the characteristics of HALVE actuators shown in this study highlight their promise for next-generation untethered soft robotic systems.

## MATERIALS AND METHODS

### Actuator materials and geometry

HALVE actuators used for characterization were made of a single pouch that was 60 mm wide by 17 mm tall. This wide aspect ratio was chosen so that the structural film edge effects were as small as possible to better match the model (*19*). The pouch’s electrode area was set at 9 mm by 59 mm and did not cover the pouch’s full width, leaving a 0.5-mm gap between the electrode and pouch seal. This gap was intended to avoid breakdown at the point of the heat seal due to the heat sealing procedure, where the P(VDF-TrFE-CTFE) layer was thinner. The top edge of the pouch was instead sealed as close as possible to the electrode without overlapping it, a requirement for forming a zipping front. The sides of the pouch section not covered by the electrodes had triangular indents to reduce the influence of edge effects (*19*). A 12.5-μm-thick BoPET film was used as the structural shell, while the electrode was made of aluminum (see Fig. 3B). The Peano-HASEL actuators used for comparison purposes had the same pouch geometry and dimensions as those described above. A 15-μm-thick BoPET film was used as the solid nonpolar dielectric and structural shell layer, while the electrodes were made from carbon ink (Electrodag 502).

The HALVE actuators used in the gripper demonstrator were made of three pouches, each measuring 35 mm in width and 15 mm in height, with electrodes covering a 34 mm–by–7.5 mm section of the pouch. A 12-μm heat-sealable BoPET film was used as the structural shell (Hostaphan RHS12). With this approach, the structural shell’s heat-sealable layer was melted, not allowing electrode traces to connect the electrode to the outside of the pouch. For this reason, 50-μm copper wires were used to connect the individual electrodes. The electrodes were made of chrome and gold. The HALVE actuators used for the robotic fish demonstrator were made of three 31 mm–by–23 mm pouches, with an area of 11.5 mm by 31 mm covered by each electrode. A 12-μm BoPVDF film was used as the structural shell. P(VDF-TrFE-CTFE) bonded to this material when melted, producing a more reliable pouch seal than BoPET alone. The electrodes were made of chrome/gold. All HASEL and HALVE actuator devices used Envirotemp FR3 from Cargill as the dielectric liquid.

### Actuator manufacturing

BoPET structural shells and aluminum electrodes were obtained by etching survival blankets (Forclaz Decathlon) with a 1% potassium hydroxide solution. The chrome-gold electrodes were produced by vapor deposition using an e-beam evaporator (VERA 450 H VTD). First, a 5-nm layer of chrome was deposited to improve adhesion, followed by a 60-nm layer of gold. The dielectric layer of the HALVE actuators was produced by blade casting a 150-μm layer of 14% wt P(VDF-TrFE-CTFE) from PiezoTech (Piezotech RT-TS) (*55*) in methyl ethyl ketone solution onto the structural shell and electrode. A TQC Sheen automatic film applicator (AB3652) with a film applicator block from Zehntner (ZUA 2000) was used. After casting, P(VDF-TrFE-CTFE) thin films were annealed at 102°C for 2 hours, resulting in a 5-μm-thick dielectric layer (see fig. S13). Further information on the casting process can be found in the Supplementary Materials.

The HALVE actuators’ oil pouches were created through heat-sealing. A standard 3D printer (Prusa MK3S) was used to print a single line of acrylonitrile butadiene styrene filament at 295°C onto a 25-μm Kapton sheet, which transferred the heat to the actuator films below. The amount of dielectric oil was chosen as 95% of the theoretical maximum cylindrical volume of the fully deformed pouch. The Peano-HASEL actuators were produced following methods described in the literature (*15*), with the only difference being that they were not sealed with the technique described above but rather by printing PLA at 220°C to activate the heat seals. More details on the heat-sealing process can be found in the Supplementary Materials.

### Dielectric measurement characterization

Dielectric displacement loop measurements of PVDF-HFP and P(VDF-TrFE-CTFE) were conducted using a Radiant Precision Premier II, equipped with a Radiant Precision 10 kV HVI-SC and a high-voltage amplifier Trek Model 609E-6. Measurements were done at ambient temperature (20°C). The P(VDF-TrFE-CTFE) samples (Fig. 2A, b) had a surface of 5 mm–by–5 mm and a thickness of 5 μm. The bottom electrode pattern was first etched onto a PET film for sample preparation. Subsequently, P(VDF-TrFE-CTFE) was blade-casted onto this PET film and electrode, using the same procedures used for the actuators. The top aluminum electrode was then sputter-coated onto the P(VDF-TrFE-CTFE) using a Safematic CCU-010. Several bipolar *D*-*E* loops were recorded at 10 Hz while incrementally increasing the applied voltage amplitude from 50 up to 1500 V.

### Fish manufacturing and measurements

The flexible body of the fish demonstrator onto which the actuators were attached was printed from black PLA with a PRUSA MK3S. The head of the fish and the ratchet mechanism were printed from Formlabs Durable Resin with a Formlabs 3 printer. The gasket that prevents water from entering the head was printed from Filaflex 60A Pro with a PRUSA MK3S+. A detailed view of the fish’s HALVE actuators and power supply is shown in fig. S10. We measured the speed and motion of the fishtail with a camera recording the fish profile from the top with a reference grid below the water tank. The energy consumption of the fish electronics unit was measured by supplying power to the fish with a DC bench power supply and recording the supply voltage and average current.

### Force-strain characterization

The actuators were characterized by a setup in which their displacement was measured, while forces of various magnitudes were applied using a set of weights. The displacement was measured using a Baumer OM70-11216521 distance sensor (see fig. S3A). Low-voltage control inputs were generated via a custom LabView Virtual Instrument and a DAQ (NI USB-6343) from National Instruments and amplified with a Trek 20/20C-HS high-voltage amplifier. To determine the quasi-static force-strain relationship of the actuators, the actuation strain was measured while applying forces between 0.25 and 5 N. The voltage signal used for the force-strain characterization was a bipolar square signal at a frequency of 0.1 Hz. The strain of four subsequent actuation cycles was recorded to obtain statistically relevant data, and the mean was calculated. To avoid transient phenomena at actuation, the data of the first second after the voltage application were not considered. This process was repeated at different actuation voltages.

### Strain rate characterization

The voltage signal used for the force-strain characterization was a bipolar square signal at a frequency of 0.1 Hz. After voltage application, the actuation time was quantified as the time required to reach the mean steady-state strain value. The strain rate was obtained by dividing the mean steady-state strain by the actuation time. To obtain statistically relevant data, each measurement was acquired four times. This process was repeated at different actuation voltages with a 0.2-N force applied to the actuators and with various weights at a constant voltage of 1100 V.

### Durability test

Durability tests were performed on single-pouch actuators with the geometry shown in Fig. 2A (a). Figure S3B shows a durability test over 2500 cycles at 1 Hz lifting a 200-g weight actuated by a bipolar 800-V signal supplied by a high-voltage laboratory power supply (TREK 20/20C-HS). The strain was recorded with the setup shown in fig. S3A. During durability tests, the actuators’ strain degraded a few percent over time with repeated actuation, as shown in fig. S3B. We observed that the amplitude of the applied field influenced this reduction in actuator strain performance. Performance degradation additionally varied across individual actuators, suggesting that manufacturing variations such as contaminants in the P(VDF-TrFE-CTFE) layer or dielectric oil, humidity levels, or degradation of the sealing lines and slight oil leakage had a part to play. Figure S3B shows a HALVE actuator constantly driven at 1100 V for 60 s. As reported by Rumley *et al.* (*49*) for PET Peano-HASEL actuators, slight charge retention can be observed.

## Supplementary Materials

### This PDF file includes:

Supplementary Materials and Methods Figs. S1 to S18 Tables S1 and S2 Legends for movies S1 to S6 References
