# Supplementary material for: Low-voltage electrohydraulic actuators for untethered robotics
Source: Sci Adv. 2024 Jan 5;10(1):eadi9319. doi: 10.1126/sciadv.adi9319 (PMC10775996; doi:10.1126/sciadv.adi9319)
Supplement: Supplementary file 1 — Supplementary Materials and Methods Figs. S1 to S18 Tables S1 and S2 Legends for movies S1 to S6 References [file sciadv.adi9319_sm.pdf]

Science Advances
AAAS

Supplementary Materials for

Low-voltage electrohydraulic actuators for untethered robotics

Stephan-Daniel Gravert *et al.*

Corresponding author: Robert K. Katzschmann, rkk@ethz.ch

Sci. Adv. **10**, eadi9319 (2024)
DOI: 10.1126/sciadv.adi9319

The PDF file includes:

Supplementary Materials and Methods
Figs. S1 to S18
Tables S1 and S2
Legends for movies S1 to S6
References

Other Supplementary Material for this manuscript includes the following:

Movies S1 to S6

Supplementary materials

Materials and methods

Structural shell and electrode fabrication

BoPET structural shell and aluminum electrodes were obtained by processing a Mylar emergency blanket (Decathlon FORCLAZ Einweg-Rettungsdecke). These blankets are composed of a thin aluminum layer sandwiched between a polyethylene terephthalate (PET) and a polyethylene (PE) layer. The PE layer of the blanket was dissolved with acetone, exposing the aluminum (see Fig. S14A). The aluminum layer was then masked with laser-cut Scotch tape (see Fig. S14B) and etched with a 2% wt. potassium hydroxide solution (see Fig. S14C). After etching, the shells were rinsed under running tap water to remove any remaining potassium hydroxide. Acetone was then used to dissolve any remnants of Scotch tape glue (see Fig. S14D). Once the masking tape was removed, the aluminum electrodes were wiped with acetone to remove any remaining adhesive, completing the structural shell and electrode fabrication.

The chrome-gold electrodes were produced by e-beam evaporation (VERA 450 H VTD). We used laser-cut acrylic masks to define the electrode positions. A $5\,\mathrm{nm}$ layer of chrome was then first deposited on the structural shell at a rate of $0.2\,\mathrm{A/s}$ to improve adhesion, followed by a $60\,\mathrm{nm}$ layer of gold deposited at a rate of $1\,\mathrm{A/s}$ (see Fig. S15). For the gripper demonstrator, the actuator's electrodes were connected to the outside of the pouch with $50\,\mathrm{\mu m}$ thick copper wire, which was connected to the electrodes with conductive copper tape before P(VDF-TrFE-CTFE) coating.

P(VDF-TrFE-CTFE) coating

The solid dielectric used for HALVE actuators was P(VDF-TrFE-CTFE) from Piezotech-Arkema (Piezotech RT-TS, P(VDF-TrFE-CTFE) terpolymer). In preparation for the P(VDF-TrFE-CTFE) coating, the actuators' shells and a glass substrate (a rectangular mirror) were wiped with ace-tone to remove dust particles and other contaminants. The actuator's structural shell and con-nected electrodes were then adhered electrode-side up to the glass substrate by laying them on an acetone pool; the acetone was then gently squeezed out from below the shells, pulling the films flat on the glass plate. A P(VDF-TrFE-CTFE) layer was then applied on top of the shells by blade casting a solution of P(VDF-TrFE-CTFE) and MEK (2-Butanone) in a $14\ \%$ to $86\ \%$ratio by weight. The equipment used for casting was a TQC sheen automatic film applicator (AB3652) and a film applicator block from Zehntner (ZUA 2000). To obtain a P(VDF-TrFE-CTFE) layer of roughly $5\ \mathrm{\mu m}$ thick, the film applicator block was set to lay a 150 $\mathrm{\mu m}$ film of P(VDF-TrFE-CTFE) and MEK solution. The coated shells were then left to air dry for 2 minutes, during which time they were taped to the substrate using masking tape to prevent sub-sequent warping in the oven. The entire process is shown in Fig. S16. The coated sheet was then annealed in an oven at $102\ ^{\circ}\mathrm{C}$ for 2 hours. A focused ion beam scanning electron micro-

scope (FIB-SEM) (ThermoScientific Helios 5 UX) was used to take a picture of a cross-section of the PET-aluminum-P(VDF-TrFE-CTFE) sandwich. Three samples (different actuators) were measured. Two were coated with 150 µm layers of P(VDF-TrFE-CTFE) and one with a 300 µm layer, which shrank to 5 µm and 11.5 µm, respectively, after evaporation and annealing (see Fig. S13A and Fig. S13B). Therefore, the ratio between cast layer thickness and final dry-layer thickness was measured to be approximately 1:30 for a casting thickness of 150 µm and approximately 1:25 for a casting thickness of 300 µm. The smaller thickness reduction ratio suggests a more porous P(VDF-TrFE-CTFE) layer for thicker P(VDF-TrFE-CTFE) coatings. Reducing the porosity might be achieved by increasing the air evaporation time before putting the samples into the oven to slow evaporation.

Pouch sealing

HALVE actuators were heat-sealed to produce the oil pouches. The actuator's two halves were first stacked by aligning the electrodes. Then the actuator was positioned on a 3D printer's bed (Prusa MK3S) between two 25 µm Kapton films. The actuator's pouches were then sealed by printing a single layer of hot filament at the sealing line location (see Fig. S17A). To seal the actuators with BoPET and Hostaphan structural shells, ABS filament was printed at a nozzle temperature of 293 °C. For the BoPVDF structural shell, PETG filament was used at a nozzle temperature of 240 °C. HASEL actuators were sealed using PLA filament at a nozzle temperature of 220 °C. The printer bed temperature was set to 30 °C for sealing.

Oil filling

Once the pouch had been sealed, it was filled with dielectric oil. The amount of oil used was 95 % of the theoretical cylindrical volume formed when the actuator was fully actuated. We tested different liquid dielectrics (Midel 7131, Shell GTL S4, Envirotemp FR3) and chose Envirotemp FR3 from Cargill. It was chosen as it has been used successfully in previous hydraulically amplified electrostatic actuators and demonstrated the best chemical compatibility with P(VDF-TrFE-CTFE) among the tested candidates. For the other oils (Midel 7131, Shell GTL S4), we observed stronger charge retention which rendered the actuators less reliable. The pouch was filled through the filling port using a syringe with a long, and thin, blunt point needle (see Fig. S17B). Once the pouch was filled, the actuator was positioned between two Kapton sheets. The filling port was then heat sealed by applying light pressure with a soldering iron set at 275 °C for BoPET and Hostaphan structural shells, 240 °C for BoPET structural shells, and 225 °C for HASEL actuators. The excess actuator's shell was then trimmed, and the mounting holes were created with a revolver hole punch.

Strain rate and specific power derivation

Peak strain rate and peak and average specific power of HALVE actuators and Peano-HASEL actuators were determined following the methodology described by Kellaris et al. (*12*). The

characterization setup shown in Fig. S3A was used to record the actuators' contraction in response to a 0.1 Hz square wave. The voltage polarity was reversed at each actuation. To smooth the measured contraction data, a Savitsky-Golay filter was applied to it (see Fig. S5A). The filter used a third-order polynomial fit and a frame length of 17. Contraction speed (see Fig. S5B) and acceleration (see Fig. S5C) were then calculated by taking the derivatives of the Savitsky-Golay polynomials. For all the calculations described below, only the period from initial movement (t_s) to reaching equilibrium contraction (t_e) was considered.

The peak strain rate was calculated as follows:

$$PeakStrainRate = \frac{v_{peak}}{L_{actuator}} \cdot 100\% \tag{7}$$

where v_{peak} is the peak contraction velocity, and $L_{actuator}$ is the actuator length.

To calculate specific power, first, the force acting on the weight hanging from the actuator was determined:

$$F_{net}(t) = m_{weight} \cdot a(t) = F_{act}(t) - F_g(t) \tag{8}$$

$$\begin{aligned} F_{act}(t) &= F_{net}(t) + F_g(t) \\ &= m_{weight} \cdot a(t) + m_{weight} \cdot a_g \\ &= m_{weight} \cdot (a(t) + a_g) \end{aligned} \tag{9}$$

where a_g is the acceleration due to gravity and m_{weight} is the mass of the hanging weight. Specific power (see Fig. S5D) was then derived by dividing the product of force and actuation speed by actuator mass:

$$P_{sp}(t) = \frac{P(t)}{m_{actuator}} = \frac{F_{act} \cdot v(t)}{m_{act}} \tag{10}$$

Average specific power was instead derived by integrating the specific power between t_s and t_e to obtain total specific work, and finally dividing by the change in time.

$$P_{sp-avg}(t) = \frac{W_{sp-total}}{t_e - t_s} \tag{11}$$

Box-constrained optimization and system identification

To integrate the area under the force-strain measurements, we used a box-constrained optimization algorithm to estimate a fitting curve. The eight relevant parameters from the force-strain equation derived by Kellaris et al. (*19*) that were optimized were width w of the pouch, thickness t of the pouch, ε_0, ε_r, applied voltage V, α_0, L_e, and L_p. We assumed physically realistic minimum and maximum values for each parameter and searched for a combination that best fits our experimental data. We additionally optimized the half-central angles α that parametrized the relation between force and strain. The parameters were normalized and clamped between 0 and 1 during optimization based on the minimum and maximum values that we set. An optimized

curve-fit – for example, $1300\,\mathrm{V}$ – can be seen in Fig. S18. Because we were interested in an analytical expression of force as a function of strain, we performed symbolic regression using PySR (*56*) on the optimized curve-fit, since this curve-fit provided us with more sampled data points for the regression than the original experimental data. Running the symbolic regression gave us several analytical expressions with increasing complexity, from which we chose Eq. 12. We observed that the range of data points given to the symbolic regression played a crucial role. The stress rapidly decayed to zero with larger strains, and if we were to sample data points uniformly on a strain domain between, for example, 0% and 100%, then most stresses would be zero, whereas the important data points at low strain would become outliers. This would be harder to symbolically regress, and, moreover, the high-strain regime was not of interest to us. Our reported results were achieved by limiting the strain domain to approximately between 1% and 10%. This does, however, imply that the predictive capabilities of the analytical expression failed at larger strains (and likely also at very small strain values).

$$F(\varepsilon) = \frac{2.81}{\sin\left(0.17(\varepsilon - 0.23)\right)} - 2.86 \tag{12}$$

HALVE Swimmer Modeling

The CAD model of the swimmer tail was loaded into the commercial numerical simulation software COMSOL (*59*). We removed the head for simplification, since for tail deformation the head is assumed to be fixed in space. We fix the connection point of the tail to the head in COMSOL and apply contractile forces on the HALVE actuator attachment point closest to the tip. We assume that the attachment point close to the head remains static. The visualization is shown in Fig. S11A.

We fit an analytical curve through our measured data points as mentioned previously and shown in Fig. S18. This acts as our force for the HALVE actuator attachment point, dependent on the current strain of the HALVE, which is based on the distance measured between the attachment points. The deformation is in free space. We modeled the PLA material as a density of $1250\,\mathrm{kg/m^3}$ and a Young's Modulus of $1 \times 10^8\,\mathrm{Pa}$. We found acceptable bending of the tip tail of around $61.4\,\mathrm{mm}$, and the fabricated swimmer shows similar results to our simulation (see Fig. S11B).

Miniature high-voltage power supply

The electrical schematic for the modular power supply, designed with ALTIUM DESIGNER software (version 22.7.1), is shown in Fig. S9. A list of the components shown in the schematic is provided in Table S2. The TinyZero from TinyCircuits was selected as the processor board. The TinyZero is equipped with a 32-bit Atmel SAMD21 ARM Cortex M0+ processor, which is integrated into a compact $20\,\mathrm{mm}$ by $20\,\mathrm{mm}$ board weighing $1.4\,\mathrm{g}$. In addition, the board includes a Bosch BMA250 3-axis accelerometer. Its small size and light weight make it a good choice for autonomous robotic systems, and it can be easily expanded with other stackable

TinyShield boards. For example, a TinyZero board can be combined with a long-range radio module or a short-range Bluetooth module for wireless communication capabilities.

The high-voltage power supplies are powered by a lithium-ion polymer battery with a capacity of ($150\,\mathrm{mA\,h}$). The battery has dimensions of $20\,\mathrm{mm}$ x $20\,\mathrm{mm}$ x $5\,\mathrm{mm}$ and weighs $3.77\,\mathrm{g}$. A step-up DC/DC converter supplies regulated power to the high-voltage DC amplifier. We chose the ultra-miniature, unipolar, regulated high voltage DC/DC converters by *HVM Technology* as the high voltage amplifiers. Fig. S8A shows the top views of two high-voltage power supplies with different power capacities. For the low-power HV amplifer ($0.1\,\mathrm{W}$), we used the *nHV Series* (nHV0510), measuring $11.4\,\mathrm{mm}$ x $8.9\,\mathrm{mm}$ x $9.4\,\mathrm{mm}$. For the more powerful HV amplifier employed in the demonstrators, we utilized a $0.5\,\mathrm{W}$ DC/DC converter from the *UMHV Series* (UMHV0510) measuring $12.7\,\mathrm{mm}$ x $12.7\,\mathrm{mm}$ x $12.7\,\mathrm{mm}$. Both power supplies can deliver up to $1\,\mathrm{kV}$ output voltage and feature a high-impedance programming input.

We used an external 12-bit digital-to-analog converter (DAC) to control the high-voltage amplifier's input voltage precisely. A level shifter was used to translate the signals from 3.3V to 5V, enabling the connection between the DAC and the microcontroller through a serial peripheral interface (SPI). Each actuation channel comprised four MOSFETs in a full H-bridge configuration: One MOSFET pair charged the actuator while the other pair discharged it. This configuration allowed for bipolar actuation of each HALVE actuator. The MOSFETs featured a maximum drain-source voltage V_{DS} of 950V and were driven by photovoltaic drivers. Each half of the H-bridge configuration in a channel was controlled through a dual P-N channel MOSFET. Consequently, a single actuation channel can be controlled using only two control pins, preventing potential short circuits (*15*). The same schematic can be replicated modularly to create multiple independent channels.

Charge retention

During testing, we noticed the same charge retention effects described by Rumley et al. (*49*). As seen in Fig. S3B strain dropped slowly during actuation and stabilized after a few seconds. When the voltage signal was turned off, the actuator remained in a non-zero actuation state due to internal fields caused by charge accumulation. Reversing the polarity mostly mitigated this effect (*49*). We saw a correlation between the contamination of the dielectric oil and the strength of charge retention. For instance, actuators with dielectric oil exposed to humidity or dust performed considerably worse. We attempted to use dielectric oils with higher moisture tolerance, such as Midel 7131 but encountered problems such as chemical incompatibility with other actuator materials.

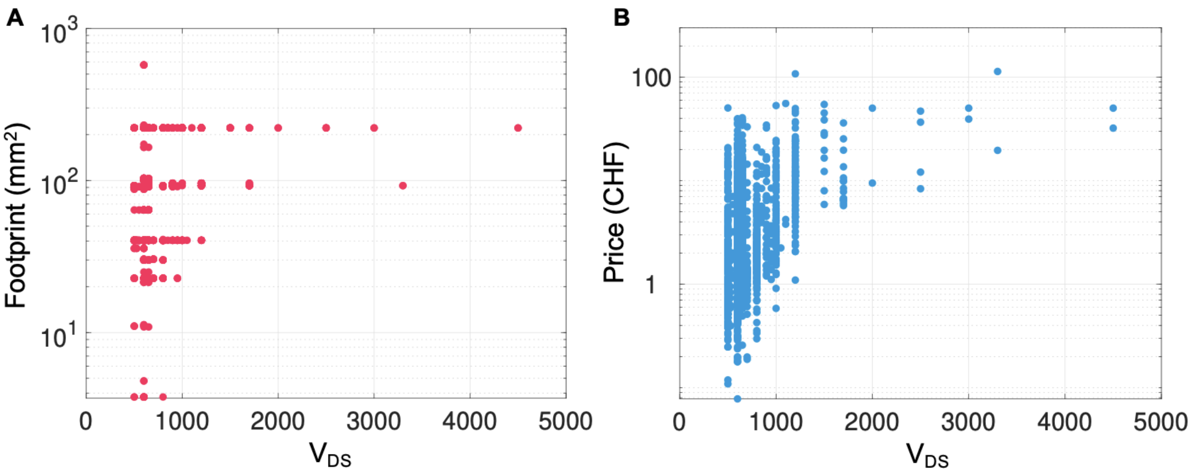

Fig. S1. Footprint and cost of high voltage MOSFETs. (**A**) 1745 data points collected from the online catalog ”Digi-Key electronics” in May 2022. All MOSFETs on the website were filtered for N-channel, surface-mounted, and a maximum drain-source voltage of $V_{DS} > 499V$. (**B**) Component price given its maximum V_{DS}.

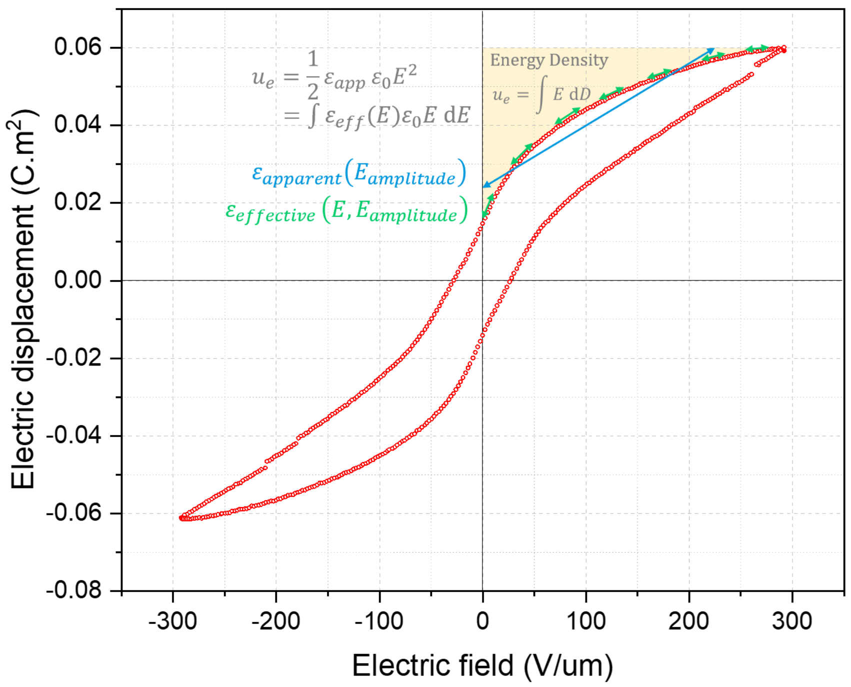

Fig. S2. D-E hysteresis cycle of P(VDF-TrFE-CTFE). Recorded at 300 V/um electric field amplitude illustrating the piecewise effective permittivity and the apparent permittivity.

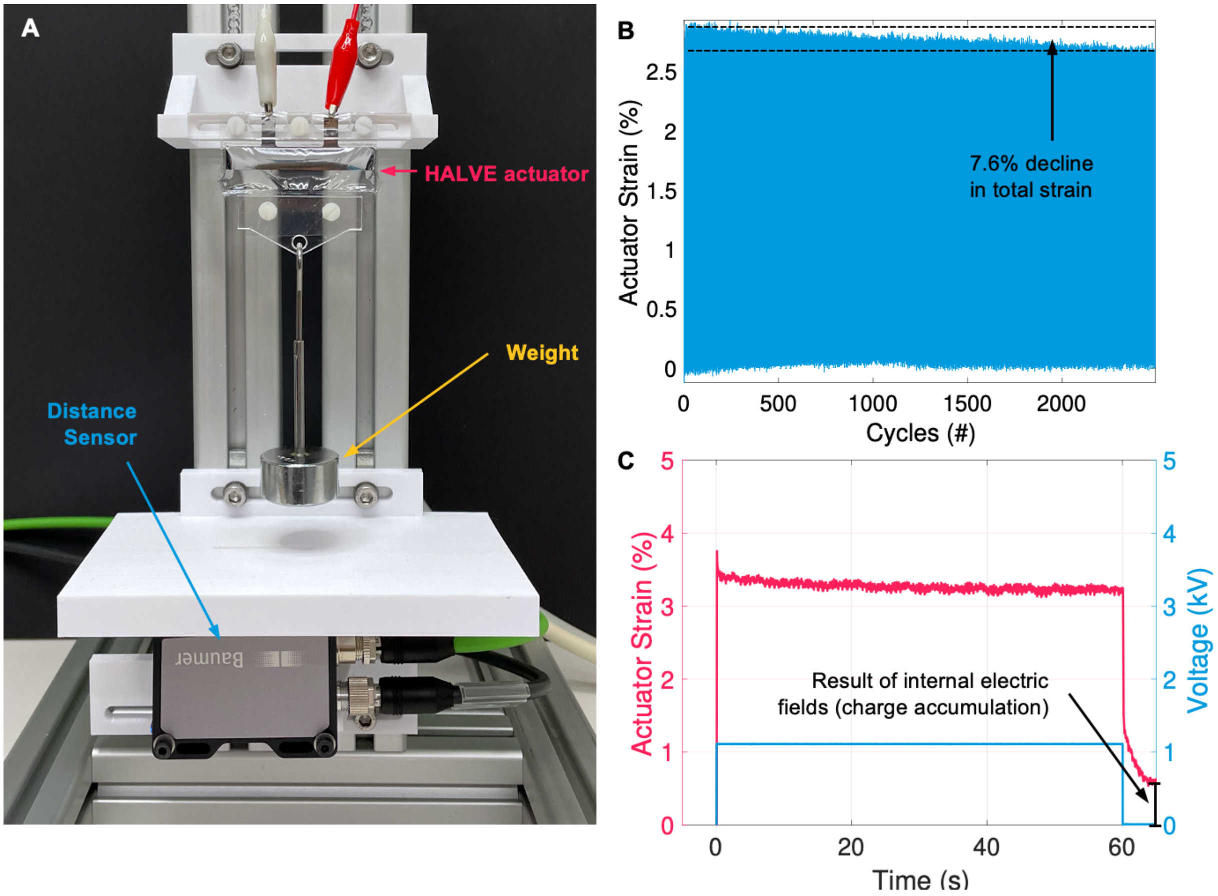

Fig. S3. Characterization setup, HALVE actuator durability test, and actuation characteristics. (**A**) Characterization setup used for actuator characterization. The characterization setup consists of an actuator plate and a laser distance sensor. Weights are attached to the actuators and strain is measured (*15*). (**B**) HALVE actuator durability test, showing strain data of a HALVE actuator (aluminum/BoPET) lifting $200\,\mathrm{g}$ with a bipolar signal of $800\,\mathrm{V}$ at $1\,\mathrm{Hz}$. A $7.6\,\%$ decline in total actuation strain can be seen after 2,500 actuation cycles. (**C**) HALVE actuator strain response to a $1100\,\mathrm{V}$ step input over 60 seconds.

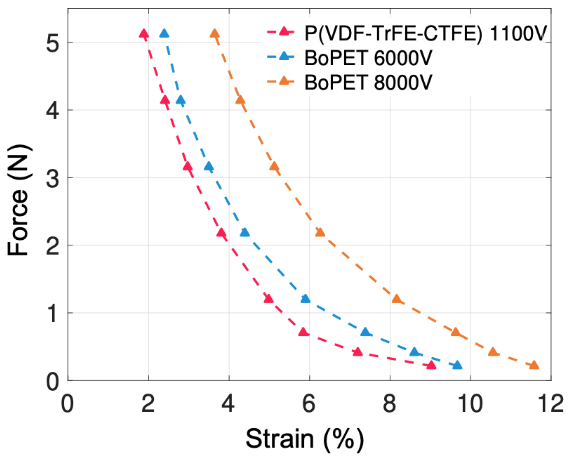

Fig. S4. Performance comparison of HALVE actuator vs. HASEL actuator. Performance comparison of a HALVE actuator made from P(VDF-TrFE-CTFE) driven at $1100\,\mathrm{V}$ vs. a HASEL actuator of the same dimension made from BoPET driven at $6000\,\mathrm{V}$ and $8000\,\mathrm{V}$.

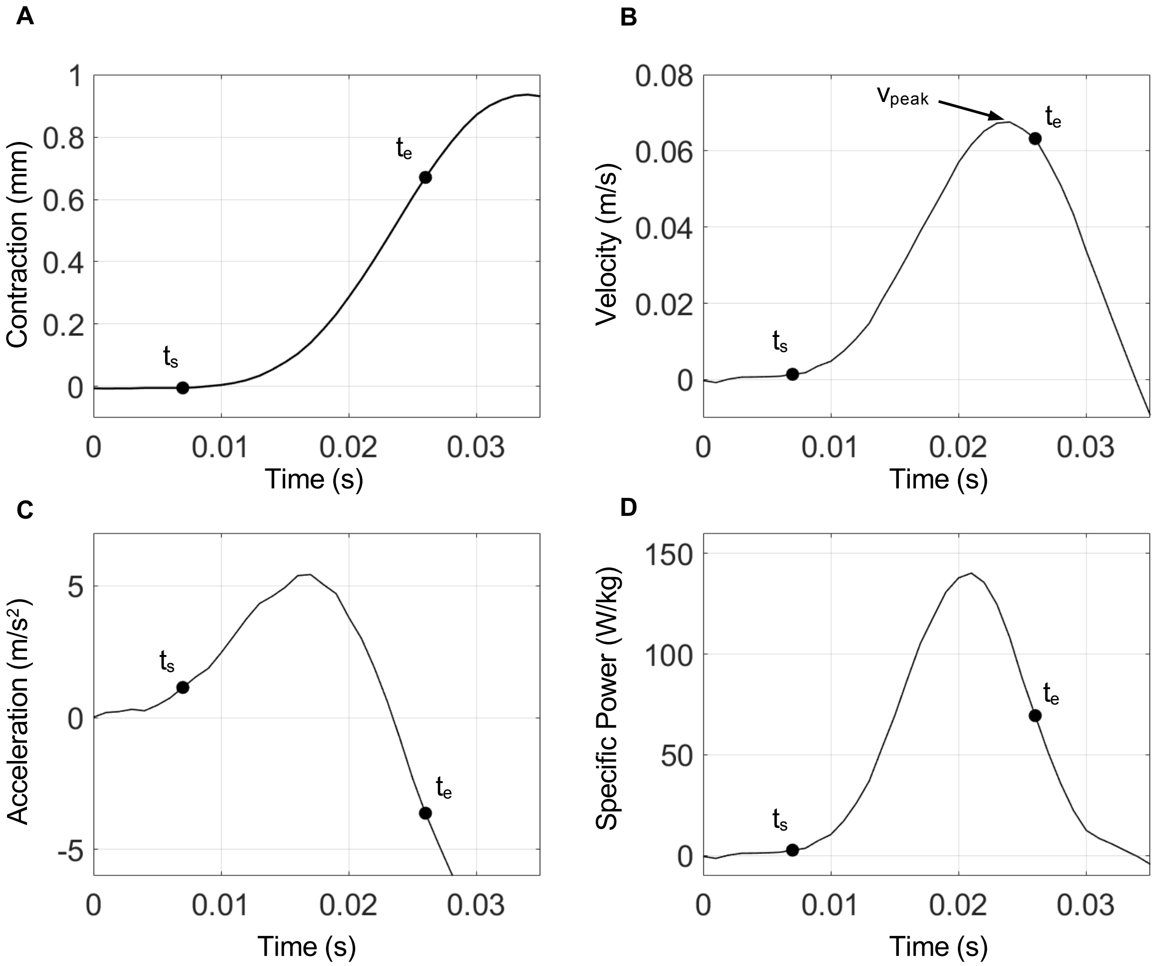

Fig. S5. Specific power calculation based on strain data from a HALVE actuator driven at 1300V while lifting 200g. (**A**) We used the same measurement method as Kellaris et al. (*12*). Measured contraction after a voltage step input was applied to the actuator. A Savitsky-Golay filter was applied to the data. (**B**) Contraction velocity curve. The maximum value of this curve was used to calculate the actuation peak strain value with Eq. 7. (**C**) Contraction acceleration curve. Both contraction velocity and acceleration were obtained by taking the derivative of the Savitsky-Golay polynomials. (**D**) Specific power curve, which was calculated using Eq. 10.

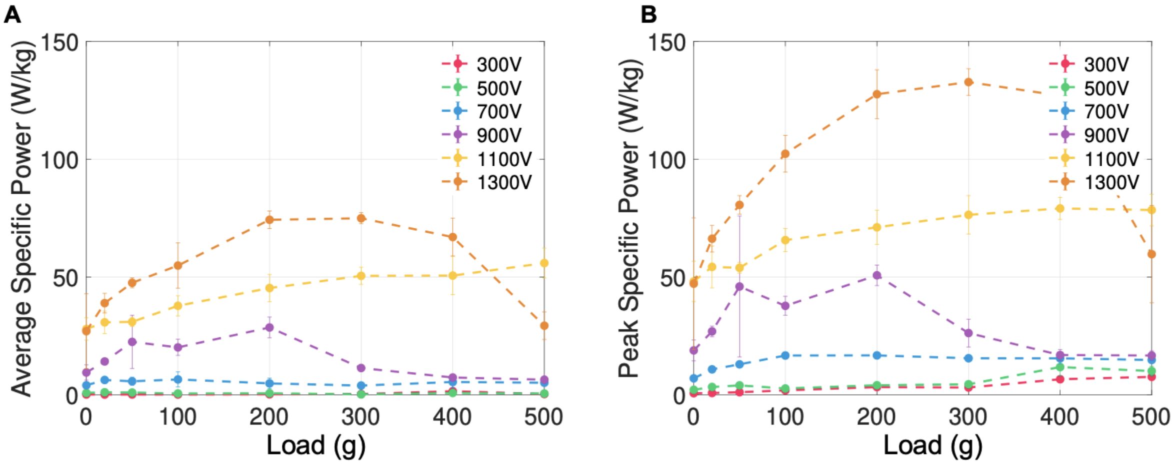

Fig. S6. Average and peak specific power of HALVE actuator devices. (**A**) Average specific power plotted against applied load for an actuation voltage range of $300\,\mathrm{V}$ to $1300\,\mathrm{V}$. (**B**) Peak specific power plotted against applied load for an actuation voltage range of $300\,\mathrm{V}$ to $1300\,\mathrm{V}$.

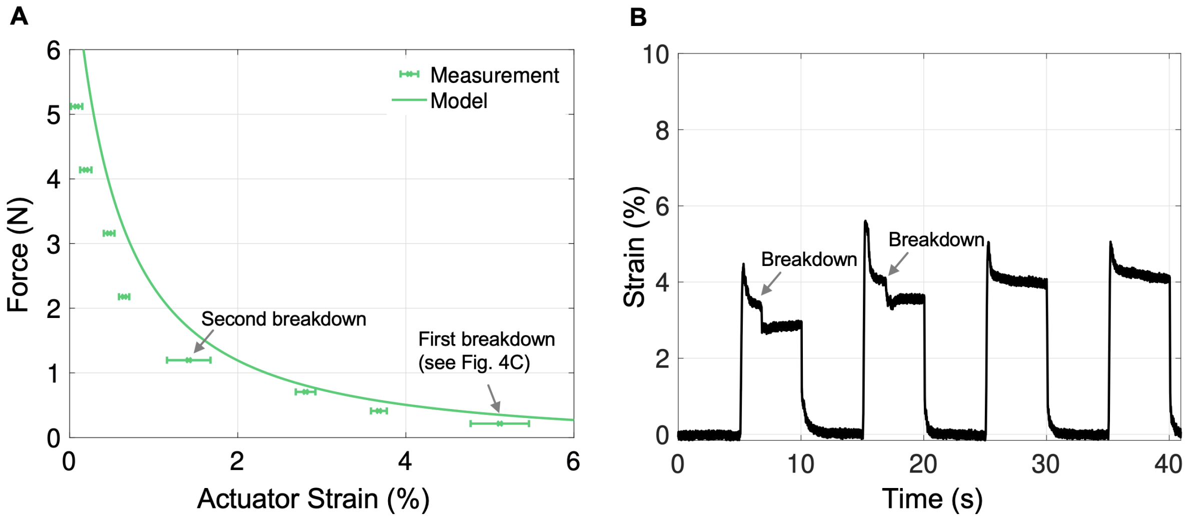

Fig. S7. Actuator with manufacturing defects undergoes multiple self-clearing events during characterization. (**A**) Full characterization of a HALVE actuator at $500\,\mathrm{V}$. The tested actuator was not produced in a clean room, which unavoidably led to the inclusion of dust particles during layer casting. These imperfections in the casted layer result in an increased frequency of breakdowns even at lower voltages. The initial breakdown occurred under a load of $22\,\mathrm{g}$, while a second breakdown occurred at a load of $122\,\mathrm{g}$. Characterization was done starting at the smallest weight up to the highest. (**B**) Aquisition of strain data at $600\,\mathrm{V}$ under a load of $42\,\mathrm{g}$ with the same actuator from panel **A** after a total of four breakdowns. Two more breakdown events can be seen with subsequent stabilization of strain performance but charge retention.

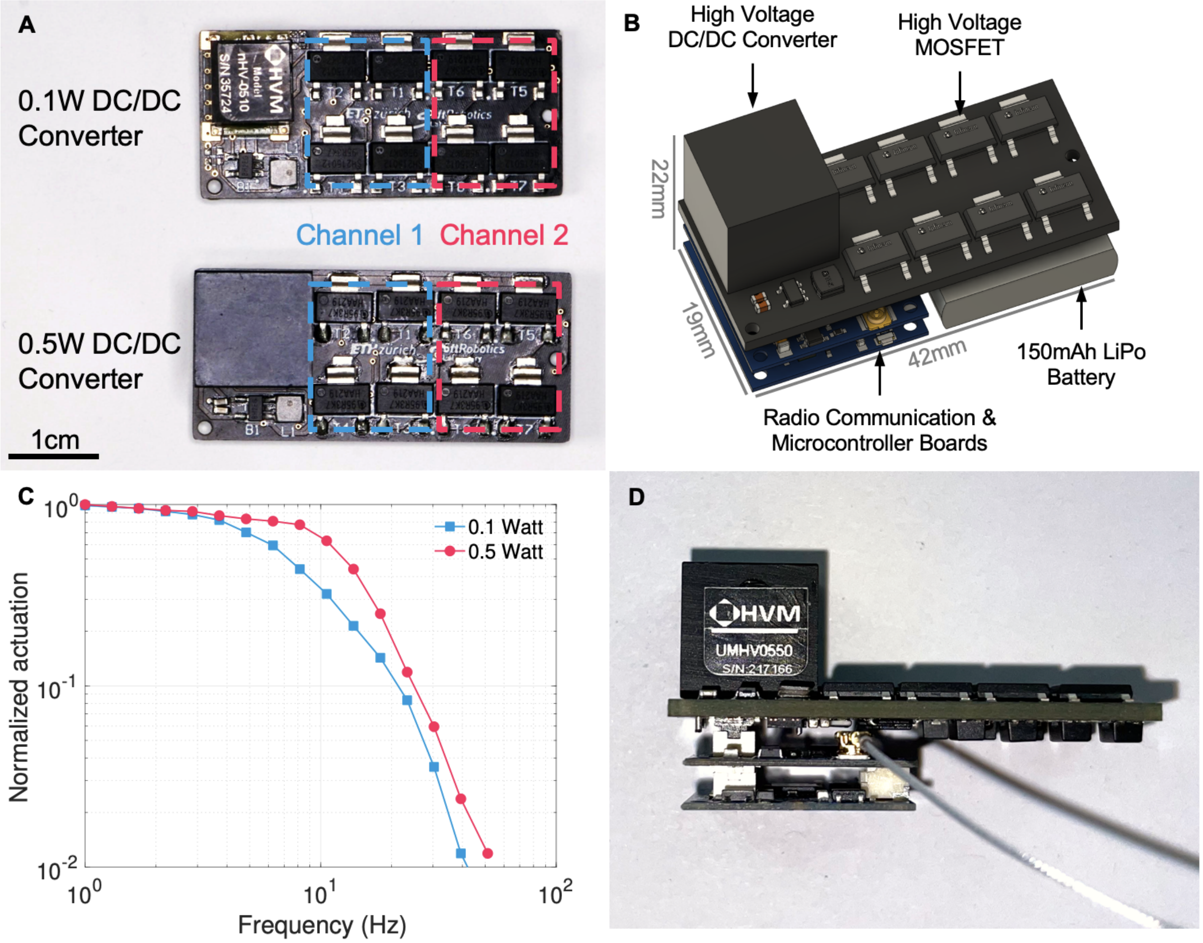

Fig. S8. Design of the compact high-voltage power supply (HVPS). (**A**) The top view of two HVPS designs with different power capabilities ($0.1\,\mathrm{W}$ and $0.5\,\mathrm{W}$) is presented. Each power supply features two bipolar channels. (**B**) Computer-aided design (CAD) representation of the 0.5 W HVPS including the battery, processor board, and radio module for communication purposes. (**C**) The frequency response of a HALVE actuator when subjected to a step input excitation signal is illustrated. (**D**) Front view of the $0.5\,\mathrm{W}$ HVPS including the processor and radio module boards.

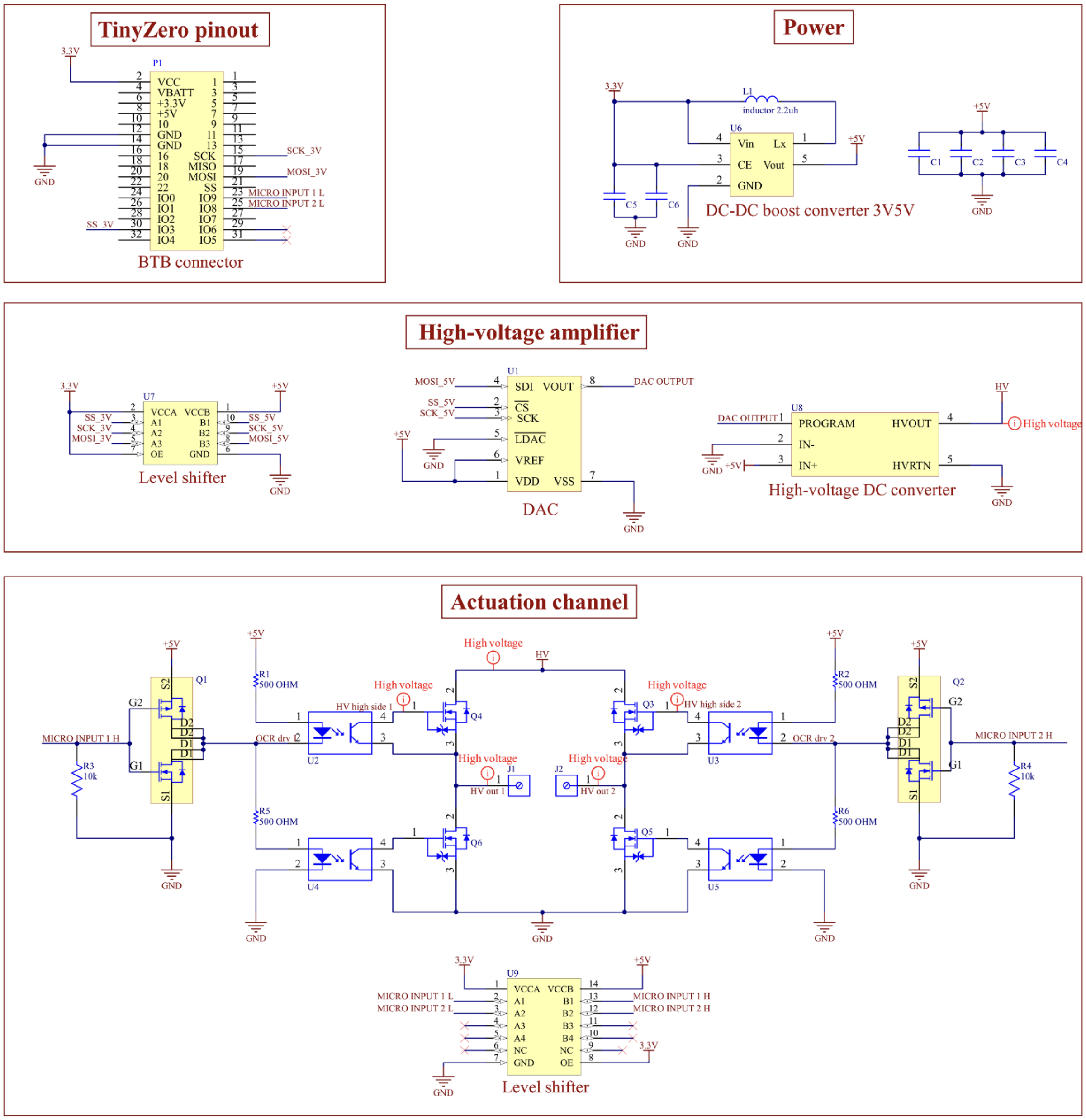

Fig. S9. Schematic of the miniature high-voltage power supply. Detailed electrical schematic of the high-voltage power supply.

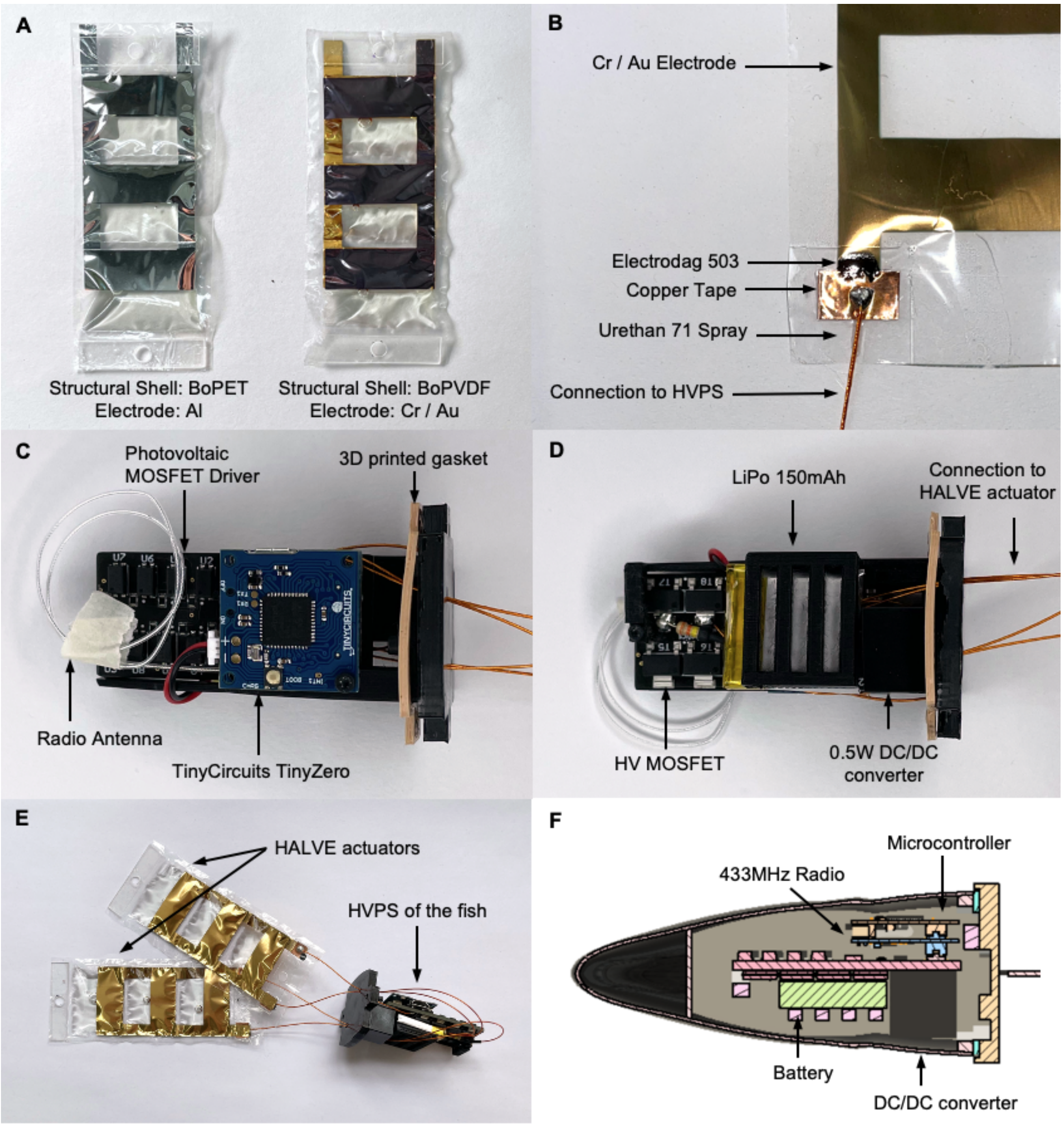

Fig. S10. HALVE actuators and power supply system for the untethered robotic fish. (**A**) HALVE actuators produced for the robotic fish. The BoPET/aluminum actuator on the left was not used for the fish because the pouch seal was deemed too unreliable for this application. (**B**) Waterproof electrical connection between a HALVE actuator electrode and a copper wire, achieved by soldering a wire to conductive copper tape, which was then adhered to the HALVE actuator. Conductive ink (Electrodag 503) was then applied to the connection, which was then waterproofed by applying Urethane 71 spray. (**C**) Top view of the two-channel robotic fish power supply. (**D**) Bottom view of the two-channel robotic fish power supply. (**E**) Connected HALVE actuators and power supply assembly used in the robotic fish. (**F**) Cross-sectional diagram of the robotic fish head.

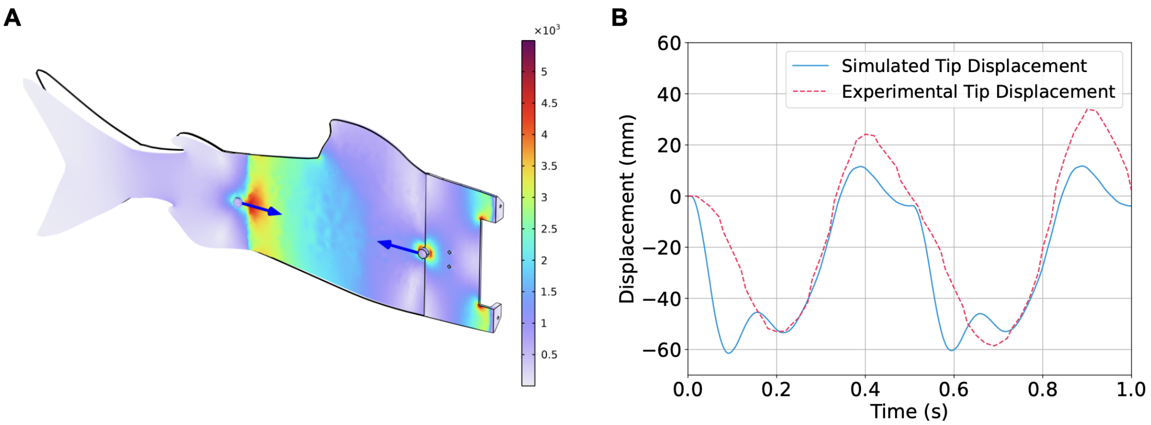

Fig. S11. COMSOL simulation of 3D swimmer actuated by HALVE actuators for flapping motion in air at 2 Hz. (**A**) Blue arrows indicate HALVE actuator forces that cause contraction. We apply forces according to the symbolically regressed force-strain curve for the HALVE actuator. Shown in color are the von Mises stresses in $\mathrm{N/m^2}$. (**B**) Comparing simulated and real deformation of the tail tip in free space.

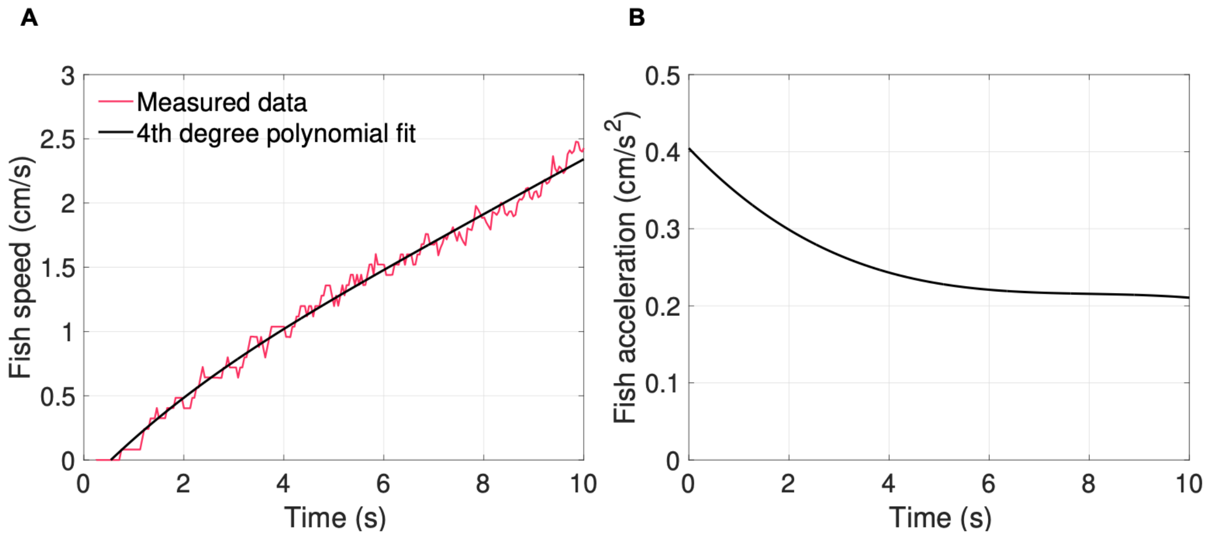

Fig. S12. Robotic fish speed characterization. (**A**) The speed of the front tip of the robotic fish is plotted vs. time for the first $10\,\mathrm{s}$ of a swim from a standstill at $2\,\mathrm{Hz}$ antagonistic actuation. (**B**) Acceleration of the robotic fish calculated as the derivative of the 4th degree polynomial fit from panel **A**.

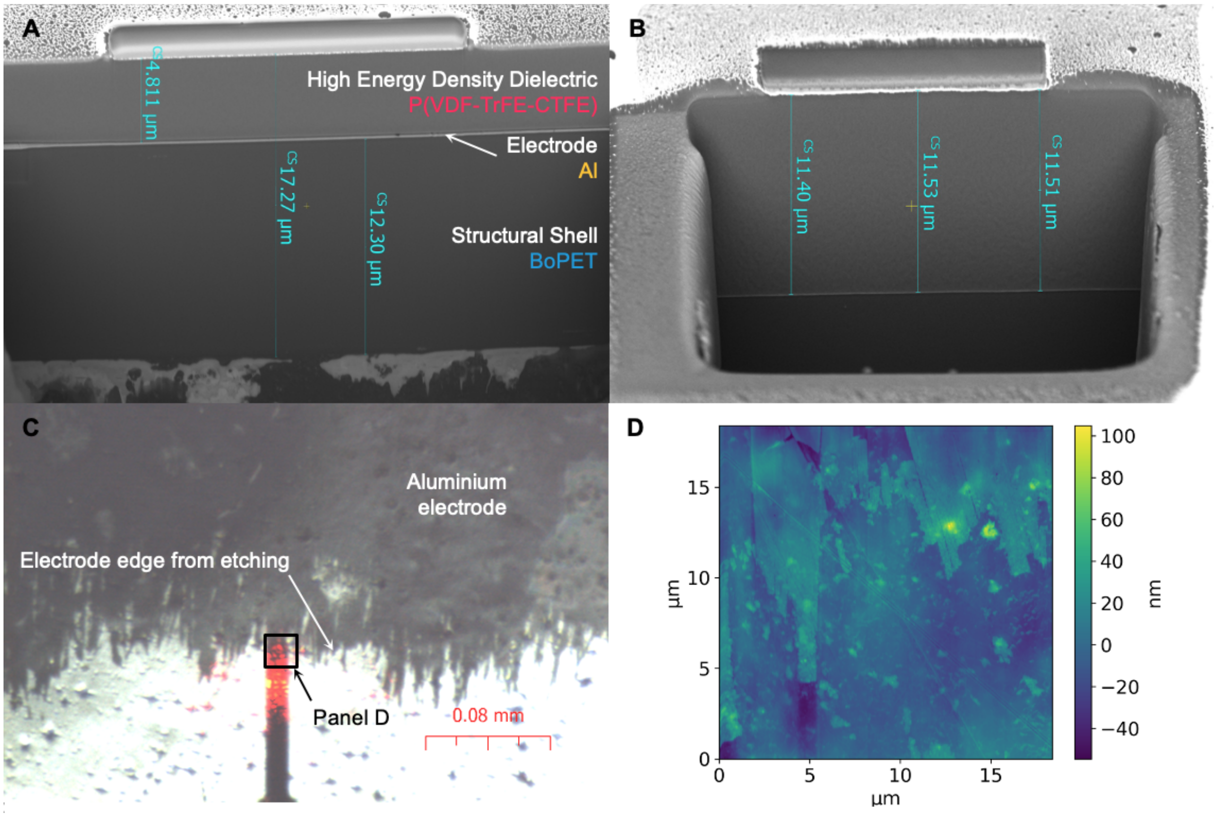

Fig. S13. Focused ion beam scanning electron microscope (FIB-SEM) and atomic force microscope (AFM) measurements. (**A**) Cross-sectional view (FIB-SEM) of a BoPET structural shell with an aluminum electrode that was blade-coated at $150\,\mu m$ thickness with P(VDF-TrFE-CTFE)/methyl ethyl ketone solution. Post-annealing, the P(VDF-TrFE-CTFE) layer reduces to around $5\,\mu m$ in thickness.(**B**) Cross-sectional view (FIB-SEM) of the same material composite structure, where $300\,\mu m$ of a mix of P(VDF-TrFE-CTFE) and methyl ethyl ketone (MEK) was coated. Post-annealing, the P(VDF-TrFE-CTFE) layer reduces to around $11.5\,\mu m$ in thickness. (**C**) Optical microscope view of parts of an aluminum electrode edge produced by etching. (**D**) AFM measurements of a small patch of the aluminum electrode surface seen in panel **C**. The surface is very consistent, with roughness at a nanometric scale ($1\,nm$-$100\,nm$).

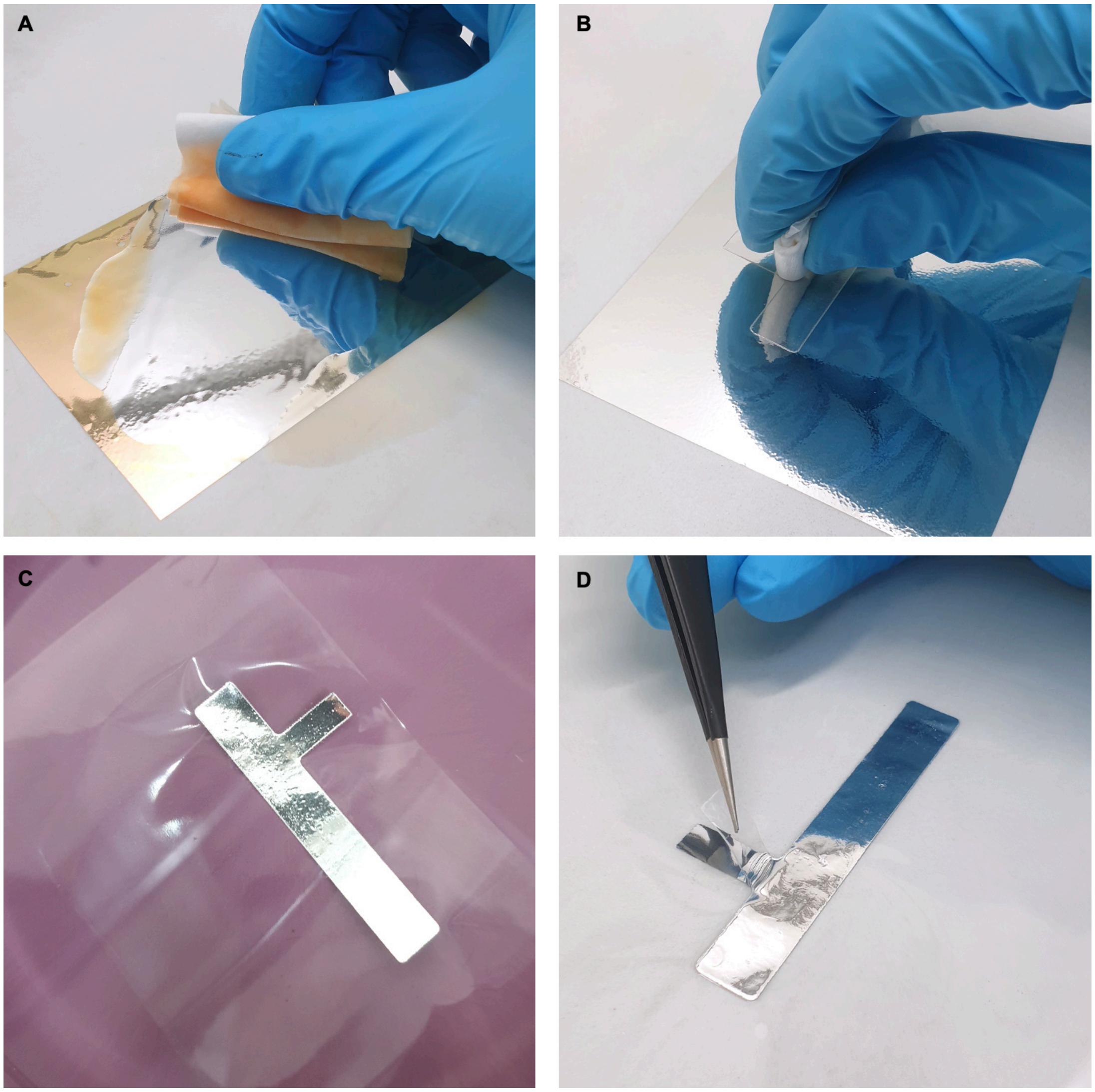

Fig. S14. Manufacturing of structural shell and electrode from a survival blanket. (**A**) Removal of the PE layer covering the aluminum using acetone. (**B**) After aluminum is exposed, a previously precisely cut masking tape is pressed onto the film. (**C**) The film is submerged into a solution of potassium hydroxide to dissolve any aluminum that is not covered by the masking tape. (**D**) Removal of the masking tape using acetone.

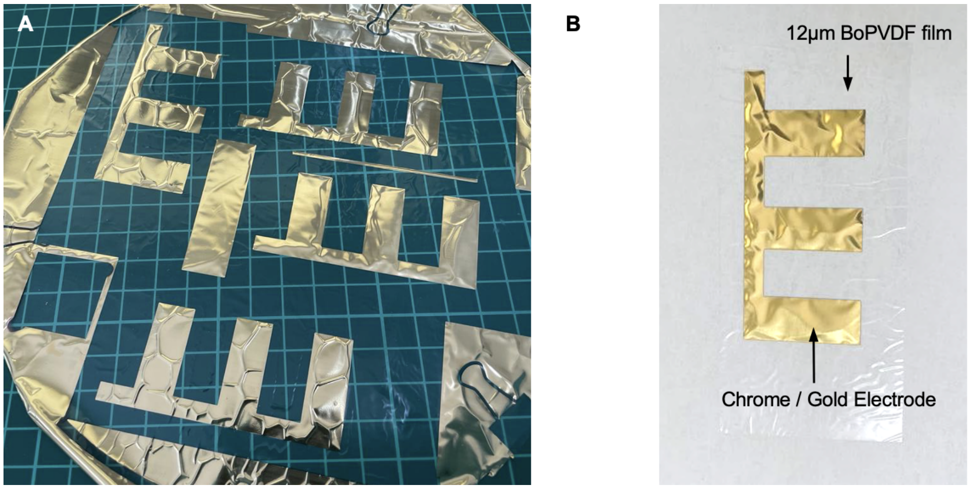

Fig. S15. Manufacturing of the BoPVDF structural shell and gold electrode with additive manufacturing for the robotic fish actuators. (**A**) A thin film of BoPVDF coated with chrome and gold using vapor deposition. An acrylic mask was used to define the electrode shapes. (**B**) Single side of an actuator after cutting, ready to be coated with the dielectric.

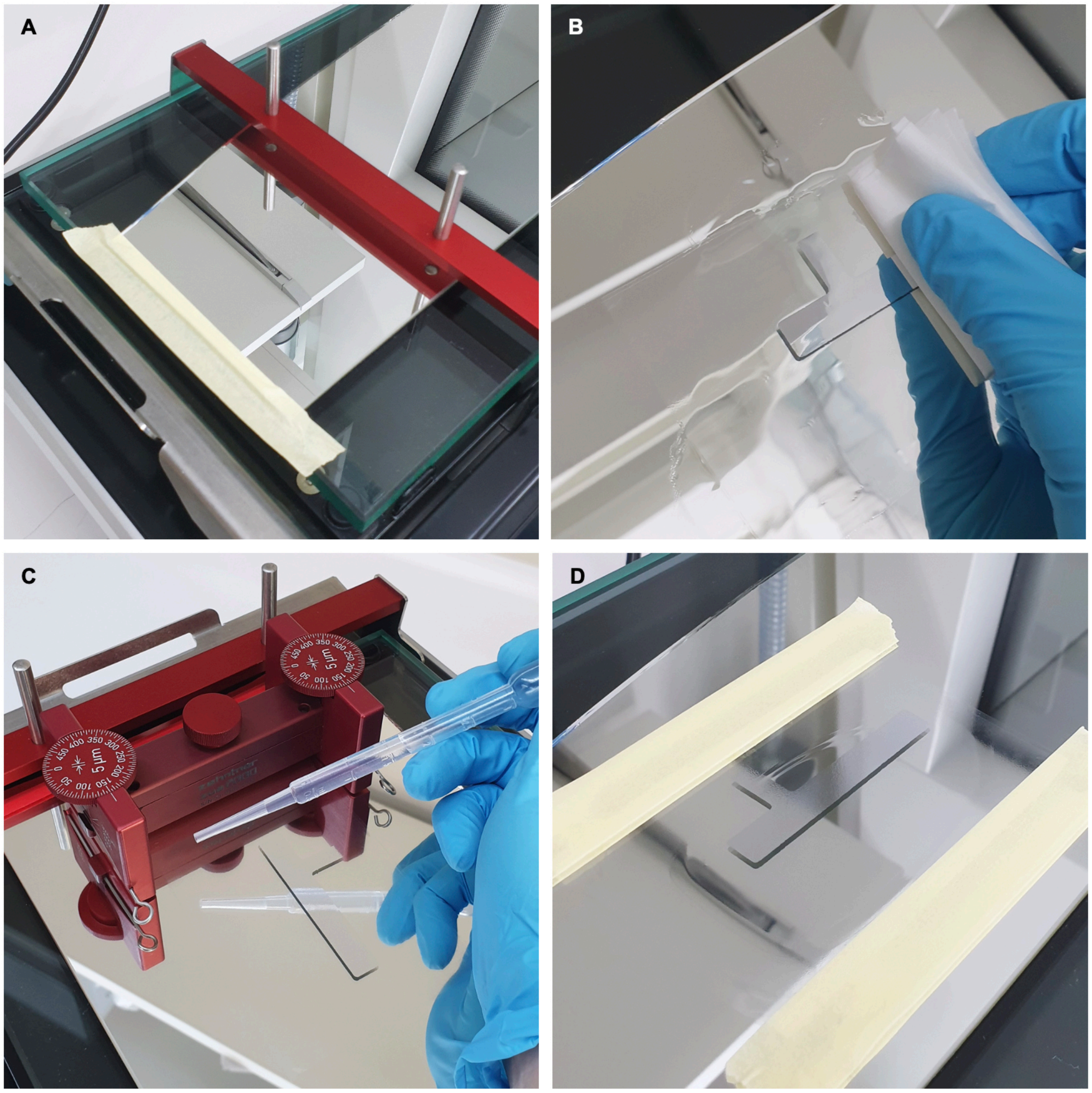

Fig. S16. Blade casting of a thin film of P(VDF-TrFE-CTFE) onto BoPET/aluminum structural shell and electrodes. (**A**) A mirror is taped to a blade coating machine. (**B**) Evaporating acetone pulls the structural shell flat onto the mirror. (**C**) A blade casting block is placed onto the applicator and P(VDF-TrFE-CTFE) solution is applied. (**D**) After application the structural shell is taped to the mirror to prevent warping during the annealing process.

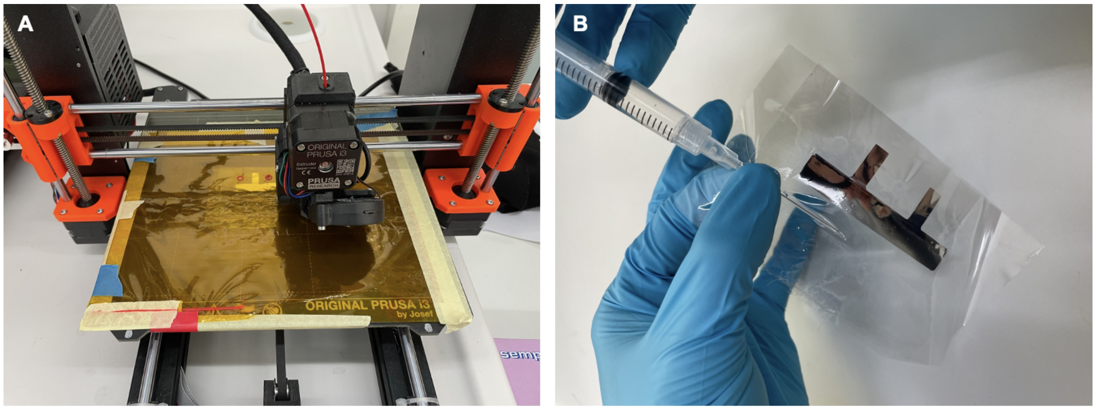

Fig. S17. Sealing and filling of the actuator. (**A**) Two coated thin films are placed between two Kapton sheets for sealing. One layer of filament is printed onto the Kapton sheet to transfer heat to the actuator's thin films. (**B**) Actuator filling with a syringe and blunt tip through a filling port.

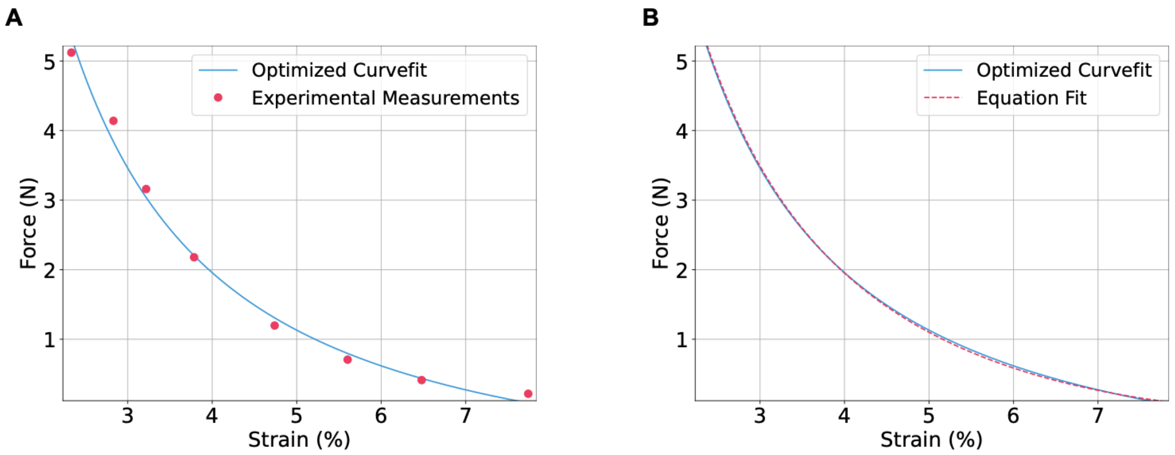

Fig. S18. Experimental actuation and regression results. (**A**) Fitting a parametric curve to experimental HALVE actuation data of $1300V$. (**B**) Symbolic regression result and equation that fits the experimentally fitted curve.

	Custom HV optocoupler (OZ100SG)	MOSFET (Infineon IPN95R3K7P7m)
Control	265 mW (*37*)	13.2 mW (*57*)
on leakage	1000 mW (*58*)	1.85 mW (*57*)
off leakage	2.5 mW (*58*)	0.95 mW (*57*)
Max voltage	10 kV (*58*)	0.95 kV (*57*)
Price	$55	$0.70
Volume	240 mm^3 (*37*)	75 mm^3 (*57*)
Weight	1.2 g (*37*)	0.11 g (*57*)

Tab. S1. Comparison between high voltage switching components. Comparison of a state-of-the-art high voltage switching optocoupler used by Mitchell et al. (*37*) to the MOSFET device used in this work.

Designator	Description	Part Number	Weight (g)
U1	12-bit Digital-to-Analog Converter	MCP4921-E/SN	0.016
U2, U3, U4, U5	Photovoltaic MOSFET Driver	APV2111V	0.043
U6	Boost Switching Regulator IC Positive Fixed 5V	RP402N501F-TR-FE	0.013
U7	Level Shifter	NTS0103GU10,115	0.052
U8 (0.1W)	High-Voltage DC-DC Converter 1000V 100mW	NHV0510	1.581
U8 (0.5W)	High-Voltage DC-DC Converter 1000V 500mW	UMHV0510	4.124
U9	Level Shifter	NTS0104BQ,115	0.016
Q1, Q2	MOSFET Array N and P-Channel	IRF7509TRPBF	0.026
Q3, Q4, Q5, Q6	Transistor MOSFET N-Channel	IPN95R3K7P7	0.115
C1, C3, C5	0.1UF Capacitor	GRM188R72A104KA35D	0.006
C2, C4, C6	10UF Capacitor	GRM188R60J106ME47D	0.006
R1, R2, R5, R6	Resistor 500 OHM	RT0603BRC07500RL	0.002
R3, R4	Resistor 10K OHM	RMCF0603JT10K0	0.002
L1	2.2UH Inductor	VLS3012HBX-2R2M	0.049

Tab. S2. Power supply components list. List of all components used for the high voltage power supply.

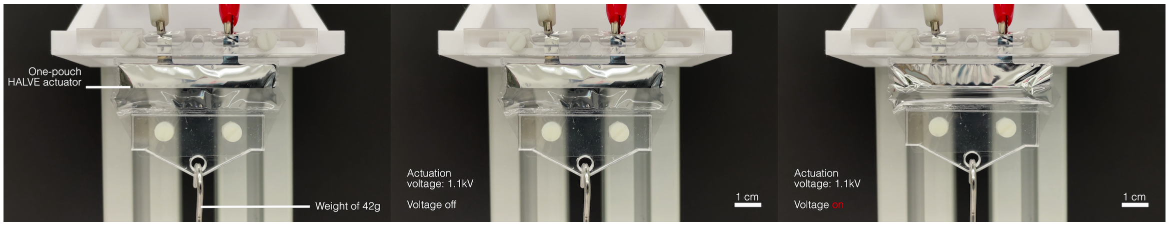

Movie S1. Demonstration of HALVE actuation.

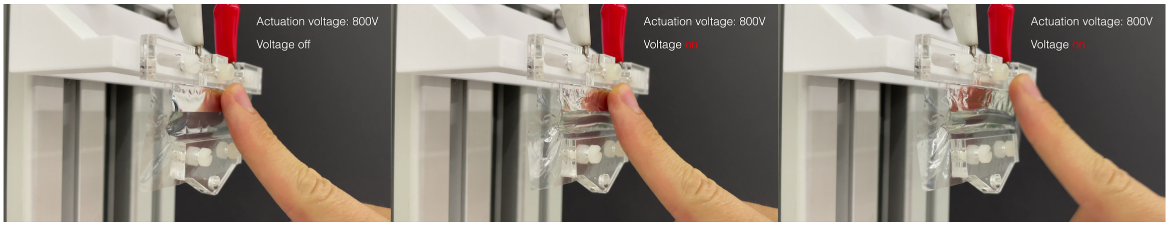

Movie S2. Touching a HALVE actuator while driven with 800V.

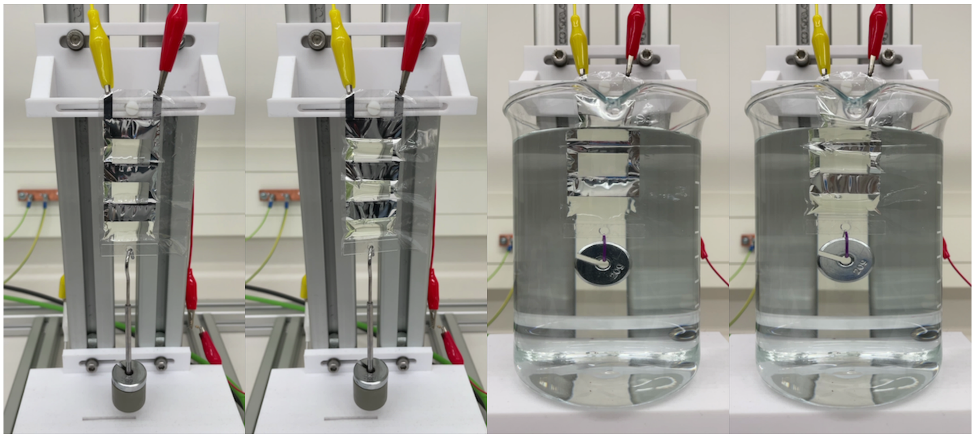

Movie S3. Three-pouch HALVE actuator operating in air and submerged in tap water.

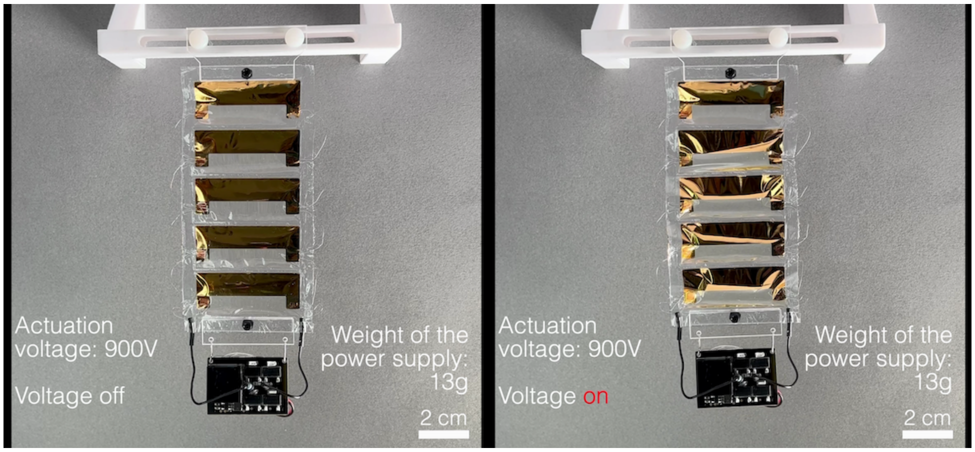

Movie S4. Untethered HALVE actuator system lifting its own power supply.

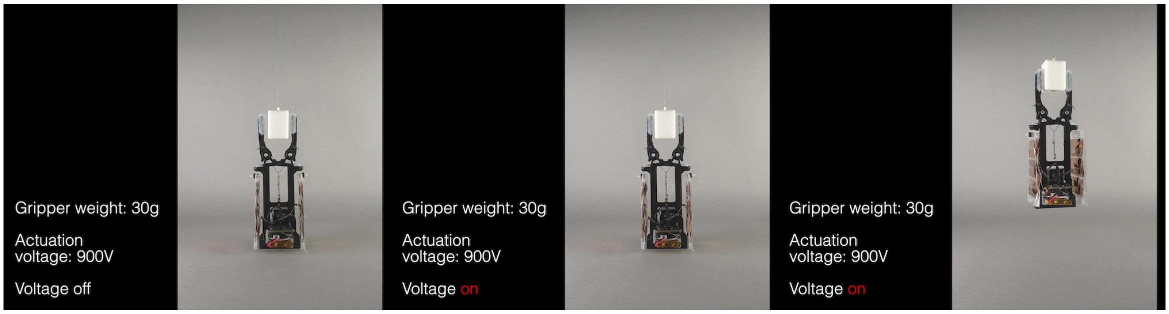

Movie S5. Untethered gripper driven by HALVE actuators grasping a PLA block.

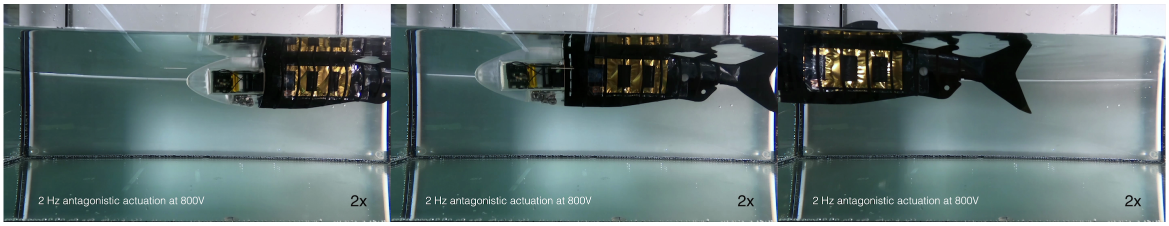

Movie S6. Untethered artificial fish driven by two antagonistic HALVE actuators swimming in tap water.

REFERENCES AND NOTES

1. F. Stella, J. Hughes, The science of soft robot design: A review of motivations, methods and enabling technologies. *Front. Robot. AI* **9** (2023).
2. E. W. Hawkes, C. Majidi, M. T. Tolley, Hard questions for soft robotics. *Sci. Robot.* **6**, eabg6049 (2021).
3. Y. Asano, T. Kozuki, S. Ookubo, M. Kawamura, S. Nakashima, T. Katayama, I. Yanokura, T. Hirose, K. Kawaharazuka, S. Makino, Y. Kakiuchi, K. Okada, M. Inaba, in *2016 IEEE-RAS 16th International Conference on Humanoid Robots* (*Humanoids*) (IEEE, 2016), pp. 876–883.
4. P. Tuffield, H. Elias, The shadow robot mimics human actions. *Int. Robot.*, **30**, 56, 60, (2003).
5. L. U. Odhner, L. P. Jentoft, M. R. Claffee, N. Corson, Y. Tenzer, R. R. Ma, M. Buehler, R. Kohout, R. D. Howe, A. M. Dollar, A compliant, underactuated hand for robust manipulation. *Int. J. Rob. Res.* **33**, 736–752 (2014).
6. A. Garcia-Garcia, B. S. Zapata-Impata, S. Orts-Escolano, P. Gil, J. Garcia-Rodriguez, in *2019 International Joint Conference on Neural Networks (IJCNN)* (IEEE, 2019), pp. 1–8.
7. S. Seok, A. Wang, Meng Yee Chuah, D. Otten, J. Lang, S. Kim, in *2013 IEEE International Conference on Robotics and Automation (ICRA)* (IEEE, 2013), pp. 3307–3312.
8. E. W. Hawkes, D. L. Christensen, A. M. Okamura, in 2016 *IEEE International Conference on Robotics and Automation (ICRA)* (IEEE, 2016), pp. 4022–4029.
9. X. Wang, S. K. Mitchell, E. H. Rumley, P. Rothemund, C. Keplinger, High-strain Peano-HASEL actuators. *Adv. Funct. Mater.* **30**, 1908821 (2020).
10. A. Hitzmann, H. Masuda, S. Ikemoto, K. Hosoda, Anthropomorphic musculoskeletal 10 degrees-of-freedom robot arm driven by pneumatic artificial muscles. *Adv. Robot.*, **32**, 865–878, 2018
11. O. Yasa, Y. Toshimitsu, M. Y. Michelis, L. S. Jones, M. Filippi, T. Buchner, R. K. Katzschmann, An overview of soft robotics, *Annu. Rev. Control, Robot. Aut.* **6**, 1–29 (2023).
12. N. Kellaris, V. Gopaluni Venkata, G. M. Smith, S. K. Mitchell, C. Keplinger, Peano-hasel actuators: Muscle-mimetic, electrohydraulic transducers that linearly contract on activation *Sci. Robot.* **3**, eaar3276 (2018).
13. E. Acome, S. K. Mitchell, T. Morrissey, M. Emmett, C. Benjamin, M. King, M. Radakovitz, C. Keplinger, Hydraulically amplified self-healing electrostatic actuators with muscle-like performance. *Science* **359**, 61–65 (2018).

14. N. Kellaris, P. Rothemund, Y. Zeng, S. K. Mitchell, G. M. Smith, K. Jayaram, C. Keplinger, Spider-inspired electrohydraulic actuators for fast, soft-actuated joints, *Adv. Sci.(Weinh)* **8**, e2100916 (2021).
15. S. K. Mitchell, X. Wang, E. Acome, T. Martin, K. Ly, N. Kellaris, V. G. Venkata, C. Keplinger, An easy-to-implement toolkit to create versatile and high-performance hasel actuators for untethered soft robots. *Adv. Sci.(Weinh)* **6**, 1900178 (2019).
16. M. Taghavi, T. Helps, J. Rossiter, Electro-ribbon actuators and electro-origami robots *Sci. Robot.* **3**, eaau9795 (2018).
17. I. D. Sîrbu, G. Moretti, G. Bortolotti, M. Bolignari, S. Diré, L. Fambri, R. Vertechy, M. Fontana, Electrostatic bellow muscle actuators and energy harvesters that stack up *Sci. Robot.* **6**, eaaz5796 (2021).
18. M. Duduta, E. Hajiesmaili, H. Zhao, R. J. Wood, D. R. Clarke, Realizing the potential of dielectric elastomer artificial muscles. *Proc. Natl. Acad. Sci. U. S. A.* **116**, 2476–2481 (2019).
19. N. Kellaris, V. G. Venkata, P. Rothemund, C. Keplinger, An analytical model for the design of peano-hasel actuators with drastically improved performance. *Extreme Mech. Lett.* **29**, 100449 (2019).
20. E. H. Rumley, D. Preninger, A. S. Shomron, P. Rothemund, F. Hartmann, M. Baumgartner, N. Kellaris, A. Stojanovic, Z. Yoder, B. Karrer, C. Keplinger, M. Kaltenbrunner, Biodegradable electrohydraulic actuators for sustainable soft robots *Sci. Adv.* **9**, eadf5551 (2023).
21. T. Helps, C. Romero, M. Taghavi, A. T. Conn, J. Rossiter, Liquid-amplified zipping actuators for micro-air vehicles with transmission-free flapping *Sci. Robot.* **7**, eabi8189 (2022).
22. M. Garrad, M. N. Zadeh, C. Romero, F. Scarpa, A. T. Conn, J. Rossiter, Design and characterisation of a muscle-mimetic dielectrophoretic ratcheting actuator. *IEEE Robot. Autom. Lett.* **7**, 3938–3944 (2022).
23. A. K. Han, S. Ji, D. Wang, M. R. Cutkosky, Haptic surface display based on miniature dielectric fluid transducers. *IEEE Robot. Autom. Lett.* **5**, 4021–4027 (2020).
24. F. Hartmann, L. Penkner, D. Danninger, N. Arnold, M. Kaltenbrunner, Soft tunable lenses based on zipping electroactive polymer actuators. *Adv. Sci.* **8**, 2003104 (2021).
25. H. Kim, J. Nam, M. Kim, K.-U. Kyung, Wide-bandwidth soft vibrotactile interface using electrohydraulic actuator for haptic steering wheel application. *IEEE Robot. Autom. Lett.* **6**, 8245–8252 (2021).

26. S. Schlatter, G. Grasso, S. Rosset, H. Shea, Inkjet printing of complex soft machines with densely integrated electrostatic actuators. *Adv. Intell. Syst.* **2**, 2000136 (2020).
27. S. Kirkman, P. Rothemund, E. Acome, C. Keplinger, Electromechanics of planar hasel actuators. *Extreme Mech. Lett.* **48**, 101408 (2021).
28. G. Moretti, M. Duranti, M. Righi, R. Vertechy, M. Fontana, in *Electroactive Polymer Actuators and Devices (EAPAD) XX*, Y. Bar-Cohen, Ed. (SPIE, 2018), vol. 10594, p. 105940W.
29. P. Rothemund, N. Kellaris, C. Keplinger, How inhomogeneous zipping increases the force output of peano-hasel actuators. *Extreme Mech. Lett.* **31**, 100542 (2019).
30. R. Hinchet, H. Shea, High force density textile electrostatic clutch. *Adv. Mater. Tech.* **5**, 1900895 (2020).
31. R. J. Hinchet, H. Shea, Glove- and sleeve-format variable-friction electrostatic clutches for kinesthetic haptics. *Adv. Intel. Syst.* **4**, 2200174 (2022).
32. E. Leroy, R. Hinchet, H. Shea, Multimode hydraulically amplified electrostatic actuators for wearable haptics. *Adv. Mater.* **32**, e2002564 (2020).
33. E. Leroy, H. Shea, Hydraulically amplified electrostatic taxels (haxels) for full body haptics. *Adv. Mater. Tech.* **8**, 2300242 (2023).
34. C. M. Keplinger, S. K. Mitchell, N. A. Kellaris, P. Rothemund, Composite layering of hydraulically amplified self-healing electrostatic transducers (WO Patent WO2020180982A1, 04 March 2020).
35. Y. J. Tan, H. Godaba, G. Chen, S. T. M. Tan, G. Wan, G. Li, P. M. Lee, Y. Cai, S. Li, R. F. Shepherd, J. S. Ho, B. C. K. Tee, A transparent, self-healing and high-κ dielectric for low-field-emission stretchable optoelectronics, *Nat. Mater.* **19**, 182–188 (2020).
36. V. Nikolic, P. Kadlec, R. Polansky, W. Guanxiang, B. C. K. Tee, in *2022 International Conference on Diagnostics in Electrical Engineering (Diagnostika)* (IEEE, 2022), pp. 1–4.
37. S. K. Mitchell, T. Martin, C. Keplinger, A pocket-sized ten-channel high voltage power supply for soft electrostatic actuators. *Adv. Mater. Tech.* **7**, 2101469 (2022).
38. X. Ji, X. Liu, V. Cacucciolo, M. Imboden, Y. Civet, A. El Haitami, S. Cantin, Y. Perriard, H. Shea, An autonomous untethered fast soft robotic insect driven by low-voltage dielectric elastomer actuators *Sci. Robot.* **4**, eaaz6451 (2019).
39. J. Madden, N. Vandesteeg, P. Anquetil, P. Madden, A. Takshi, R. Pytel, S. Lafontaine, P. Wieringa, I. Hunter, Artificial muscle technology: Physical principles and naval prospects. *IEEE J. Ocean. Eng.* **29**, 706–728 (2004).

40. I. W. Hunter, S. Lafontaine, in *Technical Digest IEEE Solid-State Sensor and Actuator Workshop* (IEEE, 1992), pp. 178–185.
41. T. Mirfakhrai, J. D. Madden, R. H. Baughman, Polymer artificial muscles. *Mater. Today* **10**, 30–38 (2007).
42. P. Rothemund, N. Kellaris, S. K. Mitchell, E. Acome, C. Keplinger, Hasel artificial muscles for a new generation of lifelike robots—Recent progress and future opportunities. *Adv. Mater.* **33**, e2003375 (2021).
43. M. Yuan, B. Li, S. Zhang, R. Rajagopalan, M. T. Lanagan, High-field dielectric properties of oriented poly(vinylidene fluoride- co -hexafluoropropylene): Structure–dielectric property relationship and implications for energy storage applications, *ACS Appl. Polymer Mater.* **2**, 1356–1368 (2020).
44. B. Chu, X. Zhou, K. Ren, B. Neese, M. Lin, Q. Wang, F. Bauer, Q. M. Zhang, A dielectric polymer with high electric energy density and fast discharge speed. *Science* **313**, 334–336 (2006).
45. S. Nishimura, S. Masuyama, G. Shimizu, C.-Y. Chen, T. Ichibayashi, J. Watanabe, Lowering of electrostatic actuator driving voltage and increasing generated force using spontaneous polarization of ferroelectric nematic liquid crystals. *Adv. Phys. Res.* **1**, 2200017 (2022).
46. B. Chu, "PVDF-based copolymers, terpolymers and their multi-component material systems for capacitor applications," thesis, The Pennsylvania State University (2008).
47. P. Rothemund, S. Kirkman, C. Keplinger, Dynamics of electrohydraulic soft actuators. *Proc. Natl. Acad. Sci. U. S. A.* **117**, 16207–16213 (2020).
48. Artimus Robotics, *Datasheet C-Series Contracting HASEL Actuator* (Artimus Robotics, 2022).
49. E. Rumley, P. Rothemund, S. Zhang, N. Kellaris, C. Keplinger, in *Electroactive Polymer Actuators and Devices (EAPAD) XXIV*, I. A. Anderson, J. D. W. Madden, H. R. Shea, Eds. (SPIE, 2022), vol. PC12042, p. PC120420B.
50. T. Wang, H.-J. Joo, S. Song, W. Hu, C. Keplinger, M. Sitti, A versatile jellyfish-like robotic platform for effective underwater propulsion and manipulation *Sci. Adv.* **9**, eadg0292 (2023).
51. S. Schlatter, P. Illenberger, S. Rosset, Peta-pico-voltron: An open-source high voltage power supply. *HardwareX* **4**, e00039 (2018).
52. S. Pourazadi, A. Shagerdmootaab, H. Chan, M. Moallem, C. Menon, On the electrical safety of dielectric elastomer actuators in proximity to the human body. *Smart Mater. Struct.* **26**, 115007 (2017).

53. X. Xie, J. Tian, X. Cao, X. Li, J. Zhang, Z. Wang, K. Ren, Ultralow-content (bi0.5na0.5) tio3–nanbo3/pvdf-hfp nanocomposites for ultrahigh-energy-density capacitor applications. *ACS Appl. Energy Mater.* **5**, 7651–7660 (2022).
54. F. M. Weiss, T. Topper, B. Osmani, S. Peters, G. Kovacs, B. Müller, Electrospraying nanometer-thin elastomer films for low-voltage dielectric actuators. *Adv. Electron. Mater.* **2**, 1500476 (2016).
55. PiezoTech, *Datasheet Relaxor-Ferroelectric and High-k Terpolymers* (Arkema S.A., 2023).
56. M. Cranmer, A. Sanchez-Gonzalez, P. Battaglia, R. Xu, K. Cranmer, D. Spergel, S. Ho, Discovering symbolic models from deep learning with inductive biases. arXiv:2006.11287 [quant-ph] (2020).
57. Infineon, *Datasheet 950V CoolMOS P7 SJ Power Device* (Infineon Technologies AG, 2018).
58. Voltage Multipliers, *Datasheet High Voltage Opto-Diode—Axial Lead* (Voltage Multipliers Inc., 2012).
59. Comsol Multiphysics, Introduction to comsol multiphysics®, COMSOL Multiphysics, Burlington, MA [accessed 9 February 2018] (1998).
